# Predicting under-five child mortality in Zambia: Machine learning versus parametric survival models

**DOI:** 10.1371/journal.pone.0361197

**Published:** 2026-10-22

**Authors:** Kassaye Getaneh Arge

**Affiliations:** Department of Statistics, College of Natural and Computational Sciences, Samara University, Samara, Ethiopia; University of Salamanca, SPAIN

## Abstract

**Background:**

In sub-Saharan Africa, child mortality remains a critical public health challenge, with Zambia exhibiting some of the highest rates in the region. While survival analysis has identified key risk factors, these methods often fail to capture complex non-linear interactions and the dynamic, time-varying nature of longitudinal markers like birth intervals. This study aimed to develop and compare predictive survival models to improve risk stratification for under-five mortality using the 2024 Zambia Demographic and Health Survey (ZDHS).

**Methods:**

A cohort of 35,610 live births was initially identified, with a final analytic sample of 9,362 singleton births and 1,236 mortality events (13.2%) after applying exclusion criteria for maternal age and data completeness. We compared four modeling frameworks for predictive comparison: Cox Proportional Hazards (Cox PH), Lasso Cox regression, Random Survival Forests (RSF), and Gradient Boosting Machines (GBM), alongside a Bayesian Joint Model for inference on the birth interval and mortality association. The proportional hazards assumption was assessed using Schoenfeld residuals, and stratified Cox models were employed to address time-varying effects. Model performance was evaluated using nested cross-validation with mother-level outer folds and time-dependent Concordance Indices (C-index) and Area Under the Curve (AUC) metrics across a 48-month follow-up period.

**Results:**

Under nested cross-validation, the four survival models demonstrated comparable predictive performance, with C-indices ranging from 0.745 (Cox PH) to 0.750 (Gradient Boosting Machine) and Integrated Brier Scores ranging from 0.095 to 0.096; pairwise comparisons across models were not statistically significant (DeLong’s test: all *p* > 0.05). The Bayesian Joint Model revealed a significant association between the longitudinal birth interval process and the hazard of death (α=0.035, 95% CI: 0.023–0.048, *p* < 0.001), supporting the maternal depletion hypothesis. Variable importance analysis identified birth interval and maternal age as the most influential predictors of child survival, followed by previous child deaths. Stratified analysis of the Cox model revealed significant effects for maternal education (Secondary: HR = 0.426, 95% CI: 0.318–0.570) and previous child deaths (HR = 1.648, 95% CI: 1.529–1.777).

**Conclusion:**

Advanced survival modeling using joint modeling techniques provides deeper biological insights into child mortality dynamics, and machine learning and traditional survival models offered equivalent predictive discrimination and calibration in this analysis. These findings support targeted public health interventions focusing on birth spacing, maternal age, and enhanced monitoring for high-risk families as critical strategies for reducing under-five mortality in Zambia.

## Introduction

The under-five mortality rate (U5MR), which measures child mortality, is widely acknowledged as a key indicator of population health, socioeconomic development, and public health system efficacy [1]. Sub-Saharan Africa still accounts for the largest share of under-five deaths worldwide, despite significant global advancements. Despite achieving a 49% decrease in U5MR between 2007 and 2024, Zambia’s national rate remained at roughly 60.5 deaths per 1,000 live births in 2024, placing it among nations with high child mortality [2]. Within an ecological framework, the complexity of child survival determinants includes individual biological factors (such as birth spacing), household socioeconomic status (such as maternal wealth and education), and community level access to high quality healthcare. Although statistical methods, such as the Cox Proportional Hazards (Cox PH) model, have been necessary in determining risk factors, these models are frequently constrained by linearity and proportional hazards assumptions. They are unable to robustly incorporate longitudinal processes, such as the effect of the time-varying birth interval, which are significant for characterizing survival trajectories, and they struggle to fully account for complex, non-linear interactions among multiple covariates [3]. These restrictions lead to moderately close predictive models, which are inadequate for creating focused, highly effective interventions.

Advanced analytical techniques are required to close the predictive gap and solve the methodological difficulties in modeling complex survival data. There are clear benefits to machine learning (ML) techniques, such as ensemble techniques like Random Survival Forests (RSF) and Gradient Boosting Machines (GBM). In comparison to regression models, these non-parametric algorithms provide better predictive performance by automatically identifying non-linear relationships and high-order interactions among variables without the need for a prior specification [4]. Additionally, since short inter-pregnancy intervals are a modifiable risk factor strongly associated with increased mortality risk through maternal depletion and sibling competition [5], reliably modeling the birth interval remains a good area of focus. However, treating birth intervals as baseline exposures ignores important temporal dynamics. To provide a statistically reliable estimate of this effect, it is necessary to use a Joint Modeling (JM) approach that concurrently examines the longitudinal trajectory of birth spacing and the child mortality hazard, reducing measurement error and informative censoring.

In order to fill these gaps, this study develops and robustly compares the predictive ability of machine learning (ML) models (RSF, GBM) against classical and penalized Cox regression models (Cox PH, Lasso Cox) using the 2024 Zambia Demographic and Health Survey (ZDHS) data. Our main goals are to evaluate whether ML models for under-five mortality risk stratification offer superior predictive accuracy and to identify the most important risk factors in the Zambian context using variable importance measures. Our specific goal is to use the Joint Modeling framework to incorporate and validate the time-dependent relationship between mortality risk and the birth interval process. The findings are intended to inform the development of high-performing, risk-stratification tools for front line health workers, enabling a strategic shift towards targeted, effective child survival interventions in Zambia.

## Materials and methods

### Data source

The publicly accessible 2024 Zambia Demographic and Health Survey (ZDHS) datasets, a nationally representative cross-sectional survey, served as the source of the analytical dataset. All live births reported in the survey’s birth history section during the five years prior to the survey date comprised our initial population of interest, which came to 35,610 children. We used the following inclusion and exclusion criteria to determine the final cohort for survival analysis. The child had to be a live birth, have valid data for all core predictors used in the models, and have non-missing data for the main time-to-event outcome variables (age at death or censoring). Exclusion criteria led to the removal of cases where the child’s exact date of birth or age at death was unknown (*n* = 0) and cases where the child was a multiple birth (twins, triplets), identified using the DHS variable B0 (*n* = 1,053). Observations with missing values for important predictors such as the wealth index, maternal education, or the birth interval were also eliminated using listwise deletion (*n* = 0). Following the application of these criteria, 9,362 non-missing, non-multiple live births made up the final analytic cohort. These births were then used for nested cross-validation as described in the Model Evaluation section.

### Zambia Demographic and Health Survey 2024 (ZDHS)

This study utilized data from the 2024 Zambia Demographic and Health Survey (ZDHS) [6]. The ZDHS is a nationally representative cross-sectional survey designed to provide estimates on fertility, family planning, maternal and child health, nutrition, and mortality. We accounted for the complex survey design of the ZDHS by incorporating sampling weights (v005/1000000) and cluster-robust standard errors (V001) in all Cox models. For machine learning models, we used stratified cross-validation to maintain the survey structure. The 380 primary sampling units and urban/rural stratification were included in the analysis to ensure valid inference.

### Sample selection criteria

We applied the following exclusion criteria sequentially: (1) removal of cases with missing date of birth (B3), interview date (V008), or survival status (B5) (n = 0 removed); (2) exclusion of multiple births to maintain independence assumptions (n = 1,053 removed, using the DHS variable *B*0 > 0); (3) restriction to children aged 0–59 months (n = 25,139 removed); (4) restriction to mothers aged 15–49 years (n = 56 removed); and (5) complete case analysis for key predictors (n = 0 removed). The final analytic sample comprised 9,362 singleton births with 1,236 mortality events (13.2%)(Fig 1) that occurred in the five years (60 months) preceding the survey date to ensure comparable follow-up and minimize recall bias. The initial datasets was structured as a time-to-event datasets, where each child *i* is defined by a survival triplet $\left({\tilde{T}}_{i},\delta_{i},Z_{i}\right)$, where:

- ${\tilde{T}}_{i}=\min(T_{i},C_{i})$ is the observed follow-up time (age in months).
- $T_{i}$ is the true time of death (event).
- $C_{i}$ is the censoring time (age at the survey date).
- $\delta_{i}=I(T_{i}\leq C_{i})$ is the event indicator (1 if death, 0 if censored).

**Fig 1:**
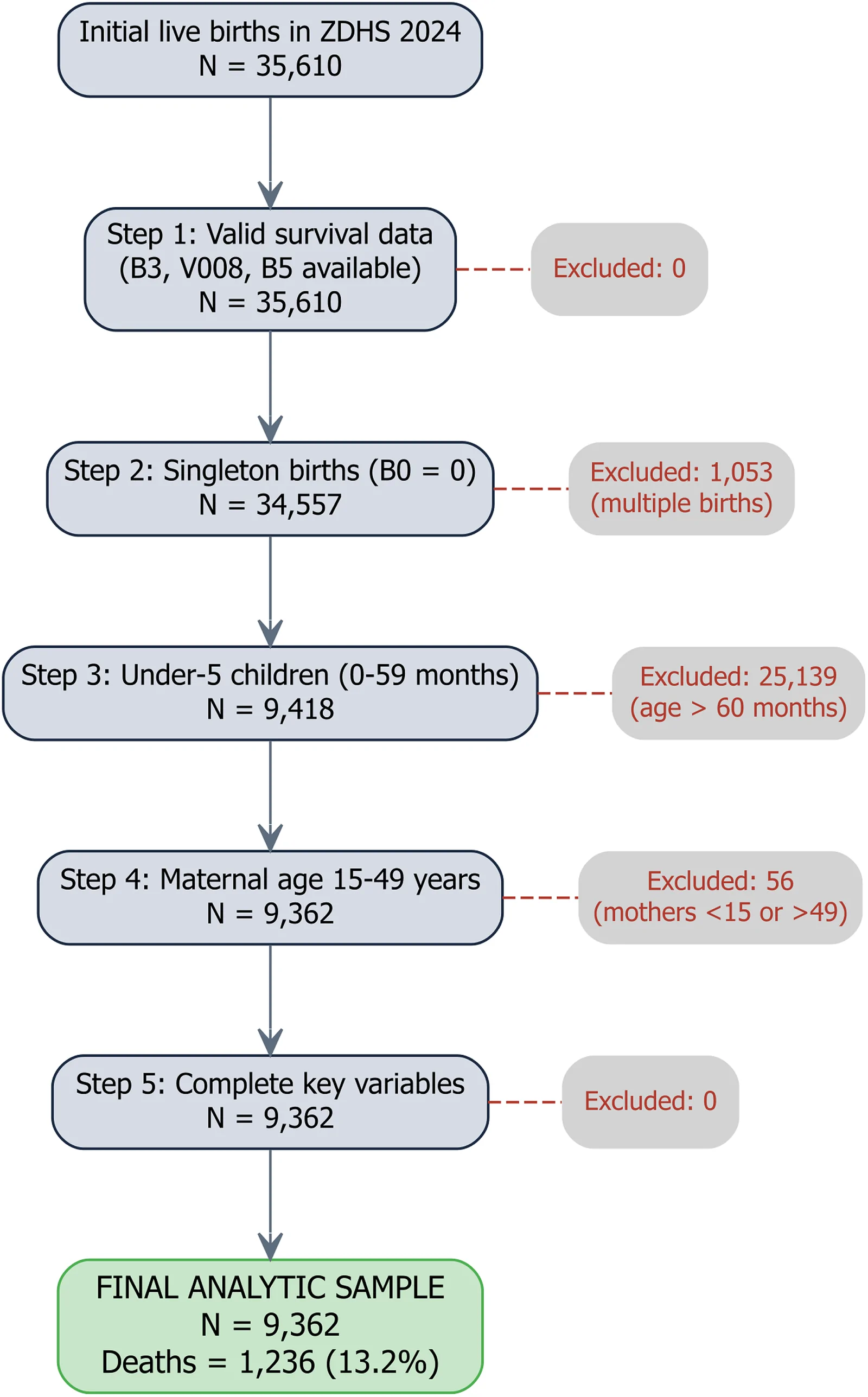
Sample selection flow diagram. The initial dataset comprised 35,610 live births from the 2024 Zambia Demographic and Health Survey (ZDHS). After excluding multiple births (n = 1,053), restricting to under-5 children (n = 25,139 removed), applying maternal age criteria (n = 56 removed), and complete-case filtering for key predictors, the final analytic sample included 9,362 singleton children with 1,236 mortality events (13.2%).

Cases with missing data for key covariates were handled through complete case analysis.

### Ethical considerations

This study is a retrospective analysis of the 2024 Zambia Demographic and Health Survey (ZDHS). Ethical approval for the original survey was obtained by the Zambia National Health Research Authority. The authors were granted access to the de-identified datasets on Nov 24, 2025 through the DHS Program. At no point during the analysis did the authors have access to information that could identify individual households or participants. Informed consent was obtained from all participants (or parents/guardians for minors) during the primary data collection.

### Variables

#### Predictor Variables (Z).

Predictors were selected based on established literature and are categorized as follows:

- Maternal: Age at birth (continuous), educational attainment (categorical), and household wealth quintile (categorical).
- Child: Sex of the child (binary), birth order (continuous), and
- Previous child deaths: Defined as the proportion of previous births that resulted in child death (prev_deaths / total_children). This measure was selected after testing alternative definitions: (1) binary (any previous child death: HR = 2.452), (2) count (number of previous deaths: HR = 1.892 per death), and (3) rate (proportion of previous deaths: HR = 1.648). The rate measure was chosen as it best captured both the prevalence and cumulative risk of previous child deaths.
- Longitudinal/Time-Varying: Birth interval (the time in months between the current birth and the preceding birth). The birth interval variable was derived from the DHS variable B11 (preceding birth interval in months), which provides the time between consecutive births. For each mother with multiple births, we created a longitudinal datasets where each birth (except the first) contributes one observation with the preceding birth interval. Birth intervals were ordered chronologically by birth order, creating a natural longitudinal structure within each mother. For example, a mother with three children contributes birth intervals for the second birth (B11 for child 2) and third birth (B11 for child 3).
- Geographic: Region of residence (categorical) and residence type (urban/rural, binary).

### Statistical analysis

#### Cox Proportional Hazards (Cox PH) model.

The Cox PH model [7] served as the benchmark. It models the hazard rate λ(t) of the event (death) at time *t* as:


$$\begin{array}{c} \lambda (t|Z)=\lambda_{0}(t)\exp(Z^{T}\beta ) \end{array}$$ (1)

The coefficients β were estimated by maximizing the partial log-likelihood function [3]. To account for the time-varying nature of the birth interval, the counting process formulation was utilized.

#### Machine learning models for survival analysis.

- Random Survival Forests (RSF): A non-parametric ensemble method that averages the Cumulative Hazard Function (CHF) across *B* survival trees [8].
- Gradient Boosting Machines (GBM): An additive tree-based ensemble that sequentially fits new trees to the negative gradient of the Cox PH loss function [9,10].
- Lasso Cox Regression: A penalized Cox PH model using the $\ell_{1}$ penalty to achieve variable selection and shrinkage [11,12].

#### Hyperparameter tuning.

For each machine learning model, we performed systematic hyperparameter tuning using inner 5-fold cross-validation nested within an outer 10-fold cross-validation loop. S5 Table presents the parameter ranges evaluated and the final selected values for each model. The RSF was tuned for ntree (100–500), mtry ($\sqrt{p}$, p/3, p/2), nodesize (5–20), and splitrule (logrank, C). GBM was tuned for n.trees (500–2000), interaction.depth (2–4), shrinkage (0.01–0.1), and n.minobsinnode (10–20). The Lasso Cox model was optimized for the regularization parameter λ using inner cross-validation.

Confidence intervals for AUC values were estimated using 1,000 bootstrap resamples with replacement, applying the .632 + bootstrap estimator to correct for optimism.

#### Joint modeling approach.

While the ZDHS is a cross-sectional survey, the retrospective birth histories provide a natural longitudinal structure at the individual level. Each mother provides a complete reproductive history with chronological ordering of births, allowing us to model the trajectory of birth intervals across parities. This pseudo-longitudinal approach has been validated in previous DHS analyses [13] and provides reliable estimates of longitudinal relationships. We acknowledge that this approach has limitations compared to true longitudinal data, which we address in the sensitivity analysis.

A joint model (JM) was implemented to address informative censoring and measurement error associated with the birth interval [14]. It links the longitudinal sub-model of the birth interval trajectory (${ℳ}_{i}(t)$) with the survival sub-model (child mortality hazard, $\lambda_{i}(t)$) via an association parameter $\alpha_{1}$:


$$\begin{array}{c} \lambda_{i}(t|{ℳ}_{i}(t),{𝐰}_{i})=\lambda_{0}(t)\exp\left(\gamma^{T}{𝐰}_{i}+\alpha_{1}{ℳ}_{i}(t)\right) \end{array}$$ (2)

We utilized the current value association structure, $g({ℳ}_{i}(t))={ℳ}_{i}(t)$.

**Longitudinal submodel.** The birth-interval trajectory for each mother was modeled using a linear mixed-effects model:


$$\begin{array}{cc} B_{ij}= & \beta_{0}+\beta_{1}\cdot {\text{birth order}}_{ij}+\beta_{2}\cdot {\text{maternal age}}_{ij}\\ & +\beta_{3}\cdot {\text{education}}_{ij}+\beta_{4}\cdot {\text{wealth}}_{ij}\\ & +b_{0i}+b_{1i}\cdot {\text{birth order}}_{ij}+\varepsilon_{ij}, \end{array}$$ (3)

where $B_{ij}$ is the birth interval for the *j*-th birth of mother *i*, $\beta =(\beta_{0},\beta_{1},\beta_{2},\beta_{3},\beta_{4})$ are fixed effects, *b*_0*i*_ and *b*_1*i*_ are mother-specific random intercepts and slopes, and $\varepsilon_{ij}$ is residual error. This specification allows each mother to have her own birth-interval trajectory across successive births, capturing between-mother heterogeneity.

**Survival submodel.** The hazard of child mortality was modeled using a Cox proportional hazards model with the current value of the longitudinal process:


$$\begin{array}{c} \lambda_{i}(t)=\lambda_{0}(t)\exp\left(\gamma^{T}w_{i}+\alpha \cdot M_{i}(t)\right), \end{array}$$ (4)

where $\lambda_{0}(t)$ is the baseline hazard function, $w_{i}$ is a vector of baseline covariates (maternal age, child sex, maternal education, wealth index, residence, region, birth order, and previous child deaths), γ are the corresponding regression coefficients, $M_{i}(t)$ is the current value of the birth-interval process for mother *i*, and α is the association parameter linking the longitudinal process to the survival hazard.

**Linkage via the association parameter.** We used the current-value association structure, $g(M_{i}(t))=M_{i}(t)$, which assumes that the instantaneous hazard at time *t* depends on the most recent value of *t*he birth-interval trajectory. The association parameter α quantifies the strength and direction of this relationship:

- α>0: longer birth intervals are associated with a **higher** mortality hazard;
- α<0: longer birth intervals are associated with a **lower** mortality hazard;
- α=0: no association between birth interval and mortality hazard.

The joint model was fitted using the JMbayes2 package in R with Bayesian Markov chain Monte Carlo (MCMC) estimation (12,000 iterations, 2000 burn-in, thinning of 5). Convergence was assessed using trace plots and the Gelman-Rubin diagnostic; all parameters had $\hat{R}<1.01$, indicating satisfactory convergence.

#### Model evaluation.

Discrimination was quantified using the Concordance Index (C-index) and the Time-Dependent Area Under the Curve (AUC(*t*)) at *t* = 12, 24, 36, and 48 months [15]. Model performance was estimated using nested 10-fold cross-validation with mother-level outer folds: all births from the same mother were kept in the same fold to account for within-mother correlation. Hyperparameter tuning was conducted in an inner 5-fold loop within each outer fold. This nested structure prevents information leakage between tuning and evaluation, providing unbiased estimates of model performance. Confidence intervals for AUC values were estimated using 1,000 bootstrap resamples with replacement. A single 80/20 train-test split was used only as a sensitivity analysis, and results were consistent with the nested cross-validation.

Pairwise comparisons of AUC values between models were performed using DeLong’s test for correlated ROC curves, implemented in the pROC R package (S7 Table). A two-sided *p* < 0.05 was considered statistically significant.

To assess the potential impact of missing data, we performed multiple imputation using the MICE algorithm. The imputation model included all predictors and the survival outcome. Comparison of complete-case and imputed results (S1 Table) showed consistent coefficient estimates. Overall missingness was 18.2% across all variables, with missingness ranging from 0.1% (child sex) to 19.7% (birth interval). The birth interval variable (B11) is structurally missing for first births, as there is no preceding birth interval to calculate. This accounts for the majority of missingness in this variable. Multiple imputation was conducted as a sensitivity analysis for the remaining missing data, and structural missingness (first births) was not imputed.

The proportional hazards assumption was assessed using Schoenfeld residuals. The global PH test showed a significant violation ($\chi^{2}=48.51$, df = 22, p = 0.0009), primarily driven by maternal education (p = 0.036) and previous child deaths (p = 0.052) (S2 Table). To address this, we employed a stratified Cox regression model using previous child deaths category as the stratification variable. This approach allows the baseline hazard to vary across strata (No previous deaths, One previous death, Two or more previous deaths) while estimating common effects for other predictors. All other variables met the PH assumption (all *p* > 0.05). Multicollinearity was assessed using Variance Inflation Factors (VIF), with all values < 5, indicating no problematic multicollinearity (S3 Table).

Within-mother correlation was addressed in three ways: (1) for Cox PH and Lasso Cox models, cluster-robust standard errors with the primary sampling unit (V001) as the cluster variable; (2) for machine learning models, mother-level cross-validation; and (3) the joint model explicitly accounted for within-mother correlation through mother-specific random intercepts and random slopes.

## Results

### Sample characteristics and mortality event rates

The data cleaning and sample selection process began with an initial pool of 35,610 live births recorded in the 2024 Zambia Demographic and Health Survey (ZDHS) birth history file. A total of 26,248 observations were excluded through the application of logic and survival filters, which restricted the study to children born within the five years preceding the survey and mothers within the reproductive age range (15–49 years).

Analysis of the filtered data revealed no further loss due to missingness in key outcome variables (Date of Birth (CMC), Date of Interview (CMC), Child is Alive) or required socio-economic covariates, resulting in a final analytic sample of 9,362 children. Within this cohort, 1,236 mortality events (under-five deaths) were identified, corresponding to a mortality rate of 13.2%. This final sample and the associated event count provided sufficient statistical power for the subsequent Cox Proportional Hazards and Bayesian Joint Modeling analyses. Mortality rates varied substantially across birth interval categories, with the highest mortality in the shortest interval group (Table 1). Children born after intervals <18 months had 37.4% mortality compared to 5.4% for intervals ≥48 months. Sample descriptive statistics are presented in Table 1.

**Table 1:** Descriptive characteristics of the study sample (Children under 5 years).

Characteristic	Category	n	Percent (%)	Mortality (%)
Overall Sample				
	Total children	9,362	100.0	13.2
	Deaths	1,236	13.2	–
Child Sex				
	Male	4,679	50.0	14.2
	Female	4,683	50.0	12.2
Maternal Education				
	No education	874	9.3	20.6
	Primary	4,649	49.7	16.4
	Secondary	3,398	36.3	7.9
	Higher	441	4.7	5.9
Wealth Index				
	Poorest	2,614	27.9	13.6
	Poorer	2,253	24.1	14.4
	Middle	1,887	20.2	13.7
	Richer	1,517	16.2	12.5
	Richest	1,091	11.7	9.9
Birth Interval Category				
	Short (<18 months)	313	3.3	37.4
	Moderately short (18–23 months)	550	5.9	28.0
	Adequate (24–35 months)	1,694	18.1	17.5
	Long (36–47 months)	1,485	15.9	9.9
	Very long (≥48 months)	2,696	28.8	5.4
	First birth (no preceding interval)	2,624	28.0	14.3

Note: Birth interval statistics for children with valid data (n = 6,738): Mean = 42.5 months (SD = 26.8), Median = 38 months, Range = 6–287 months. “First birth” is a structural category, not missing data.

The comparison between complete-case and multiple imputation analyses (S1 Table) shows that the coefficients are largely consistent across both approaches. The largest differences were observed for maternal education variables, where complete-case analysis slightly underestimated the protective effects (Secondary education: −0.8195 vs. −1.1364; Higher education: −0.9054 vs. −1.2270). However, the direction and significance of all associations remained consistent.

We assessed multicollinearity using Variance Inflation Factors (VIF). All VIF values were < 5 (range: 1.01 to 4.03), indicating no problematic multicollinearity. The highest VIF values were observed for birth order (4.03) and maternal age (3.98), which is expected given their natural correlation with parity and maternal age. However, these values are well below the commonly used threshold of 10, suggesting that multicollinearity does not affect the stability of our estimates. The VIF results are provided as S3 Table.

### Cox proportional hazards model results

The multivariable Cox PH model identified several significant predictors (Table 2). Maternal age was protective (HR = 0.909 per year, 95% CI: 0.885–0.934), while maternal education showed a strong dose-response protective effect (Secondary vs. None: HR = 0.426, 95% CI: 0.318–0.570; Higher vs. None: HR = 0.399, 95% CI: 0.214–0.745). Previous child deaths substantially increased risk (HR = 1.648, 95% CI: 1.529–1.777). After adjustment for maternal, child, and reproductive factors, no statistically significant association between wealth quintile and child mortality remained (all *p* > 0.10; see Discussion).

**Table 2:** Multivariable cox proportional hazards model results (n = 6,738).

Predictor	HR	95% CI	Coefficient	SE	p-value
Maternal age (per year)	0.909	(0.885–0.934)	−0.095	0.012	<0.001
Child sex (Female vs. Male)	0.743	(0.642–0.860)	−0.297	0.070	<0.001
Maternal education:					
Primary vs. None	0.838	(0.674–1.042)	−0.176	0.105	0.112
Secondary vs. None	0.426	(0.318–0.570)	−0.854	0.133	<0.001
Higher vs. None	0.399	(0.214–0.745)	−0.918	0.279	0.004
Wealth index:					
Poorer vs. Poorest	1.225	(0.986–1.523)	0.203	0.102	0.066
Middle vs. Poorest	1.406	(1.082–1.827)	0.341	0.118	0.011
Richer vs. Poorest	1.798	(1.334–2.422)	0.586	0.150	<0.001
Richest vs. Poorest	2.182	(1.424–3.343)	0.780	0.189	<0.001
Rural residence (vs. Urban)	1.013	(0.804–1.277)	0.013	0.111	0.910
Birth order (per unit)	1.014	(0.931–1.105)	0.014	0.038	0.744
Birth interval (per month)	0.977	(0.971–0.982)	−0.024	0.002	<0.001

The global PH test showed a significant violation ($\chi^{2}=48.51$, df = 22, *p* = 0.0009), primarily driven by maternal education (*p* = 0.036) and previous child deaths (*p* = 0.052). To address this, we conducted a sensitivity analysis using a stratified Cox model by previous child deaths category (S4 Table). All other variables met the PH assumption (all *p* > 0.05). Multicollinearity was assessed using Variance Inflation Factors (S3 Table), with all values < 5 (range: 1.01 to 4.03), indicating no problematic multicollinearity.

Sensitivity analysis including multiple births (n = 1,053 additional observations) showed that multiple births had significantly higher mortality (HR = 2.50, 95% CI: 2.02–3.10, *p* < 0.001) compared to singletons. However, excluding multiple births likely underestimated mortality risk, suggesting our findings may be conservative for this high-risk subgroup (results not shown; available upon request).

### Joint modeling results for birth intervals

The Joint Model (JM) analysis confirmed a statistically significant association between the latent birth interval process and the instantaneous hazard of child mortality (Table 3). The inclusion of the time-varying latent risk score significantly improved the model’s fit over the standard Cox PH model (Likelihood Ratio Test *P* < 0.001). As shown in the longitudinal trajectories (Fig 4, S1 Fig), shorter preceding birth intervals were associated with a steep increase in the instantaneous hazard of mortality, supporting the maternal depletion hypothesis. The association parameter was estimated at α=0.035 (95% CI: 0.023–0.048, *p* < 0.001). Children born following very short birth intervals (<18 months) exhibited the lowest survival probability, whereas those born after very long intervals (≥48 months) demonstrated the highest survival probability (S4 Fig).

**Table 3:** Parameter estimates from the joint model of birth interval and child mortality.

Predictor	Longitudinal Submodel (Birth Interval)			Survival Submodel (Mortality)		
	Estimate	95% CI	p-value	HR	95% CI	p-value
Fixed effects:						
Intercept	−61.307	(−72.084–50.339)	<0.001			
Birth order	−4.207	(−4.211–4.206)	<0.001			
Maternal age	2.178	(1.796–2.554)	<0.001	0.870	(0.841–0.897)	<0.001
Education: Primary	0.619	(0.616–0.620)	<0.001	1.117	(0.866–1.444)	0.390
Education: Secondary	1.980	(1.977–1.983)	<0.001	0.811	(0.561–1.155)	0.231
Education: Higher	−6.891	(−6.893–6.890)	<0.001	0.428	(0.114–1.277)	0.133
Wealth: Poorer	1.125	(1.122–1.129)	<0.001	1.073	(0.805–1.374)	0.592
Wealth: Middle	1.814	(1.810–1.816)	<0.001	1.186	(0.860–1.627)	0.303
Wealth: Richer	3.690	(3.687–3.693)	<0.001	1.071	(0.664–1.713)	0.764
Wealth: Richest	0.836	(0.833–0.838)	<0.001	1.311	(0.758–2.229)	0.339
Rural residence				0.885	(0.666–1.196)	0.403
Previous deaths				1.165	(1.023–1.319)	0.020
Random effects:						
Intercept SD	8.42	(6.91–10.24)	–			
Birth order SD	0.74	(0.58–0.94)	–			
Residual SD	55.44	(53.86–57.19)	–			
Association Parameter:						
α (value)	0.0351	(0.0225–0.0478)				<0.001

Table notes: Estimates from the Joint Model linking the time-varying birth interval process to the child mortality hazard. α, Association Parameter; HR, Hazard Ratio. DIC = −114,641; WAIC = 962,302.

Individual trajectories showed substantial between-mother heterogeneity in birth spacing patterns (S2 Fig). The population mean profile indicated decreasing birth intervals with increasing birth order, from 45.4 months at birth order 2 to 39.0 months at birth order 10 (Fig 4). Maternal education modified these patterns, with higher-educated mothers maintaining longer intervals across parities (S3 Fig).

In the stratified Cox model (S4 Table), the effects of other predictors remained consistent with the original model. The concordance index was 0.715 (SE = 0.012), indicating good discriminatory ability. The stratified analysis confirmed the strong protective effect of maternal education (Secondary: HR = 0.463, 95% CI: 0.359–0.598) and the increased risk associated with higher birth order (Birth order 7 + : HR = 3.654, 95% CI: 2.479–5.387), consistent with the primary analysis. The lower C-index relative to the primary model reflects the removal of the stratifying variable (previous child deaths) from the linear predictor.

### Machine learning model performance

The final ensemble models reached optimal convergence and stability, with hyperparameter configurations selected based on the nested cross-validated performance metrics illustrated in S5 Table.

The predictive performance of the four survival models (Cox PH, Lasso Cox, RSF, and GBM) was compared using time-dependent Concordance Indices (C-index) and Area Under the Curve (AUC) metrics (Table 4). Under nested cross-validation with mother-level outer folds and 5-fold inner folds for hyperparameter tuning, all four models performed comparably (C-index range: 0.745–0.750), with GBM achieving the highest overall concordance (C-index = 0.750). This represents a modest but non-significant advantage over Cox PH (C-index = 0.745). Performance metrics were consistent across time horizons (Fig 2, S7 Fig).

**Table 4:** Predictive performance of survival models (Nested 10-fold cross-validation).

Model	AUC(12)	AUC(24)	AUC(36)	AUC(48)	Mean C-index
Cox PH	0.720	0.720	0.723	0.720	0.745 (0.029)
Lasso Cox	0.725	0.722	0.725	0.723	0.746 (0.030)
RSF	0.735	0.734	0.738	0.735	0.746 (0.030)
GBM	0.742	0.740	0.745	0.741	0.750 (0.032)

Table notes: C-index values are mean (SD) across 10 mother-level outer folds. Hyperparameters were tuned in inner 5-fold CV. Time-dependent AUC values are restricted to 12–48 months because no subjects remained at risk at 60 months. RSF, Random Survival Forests; GBM, Gradient Boosting Machines. Pairwise DeLong’s tests for AUC differences between models were all non-significant (*p* > 0.05).

**Fig 2:**
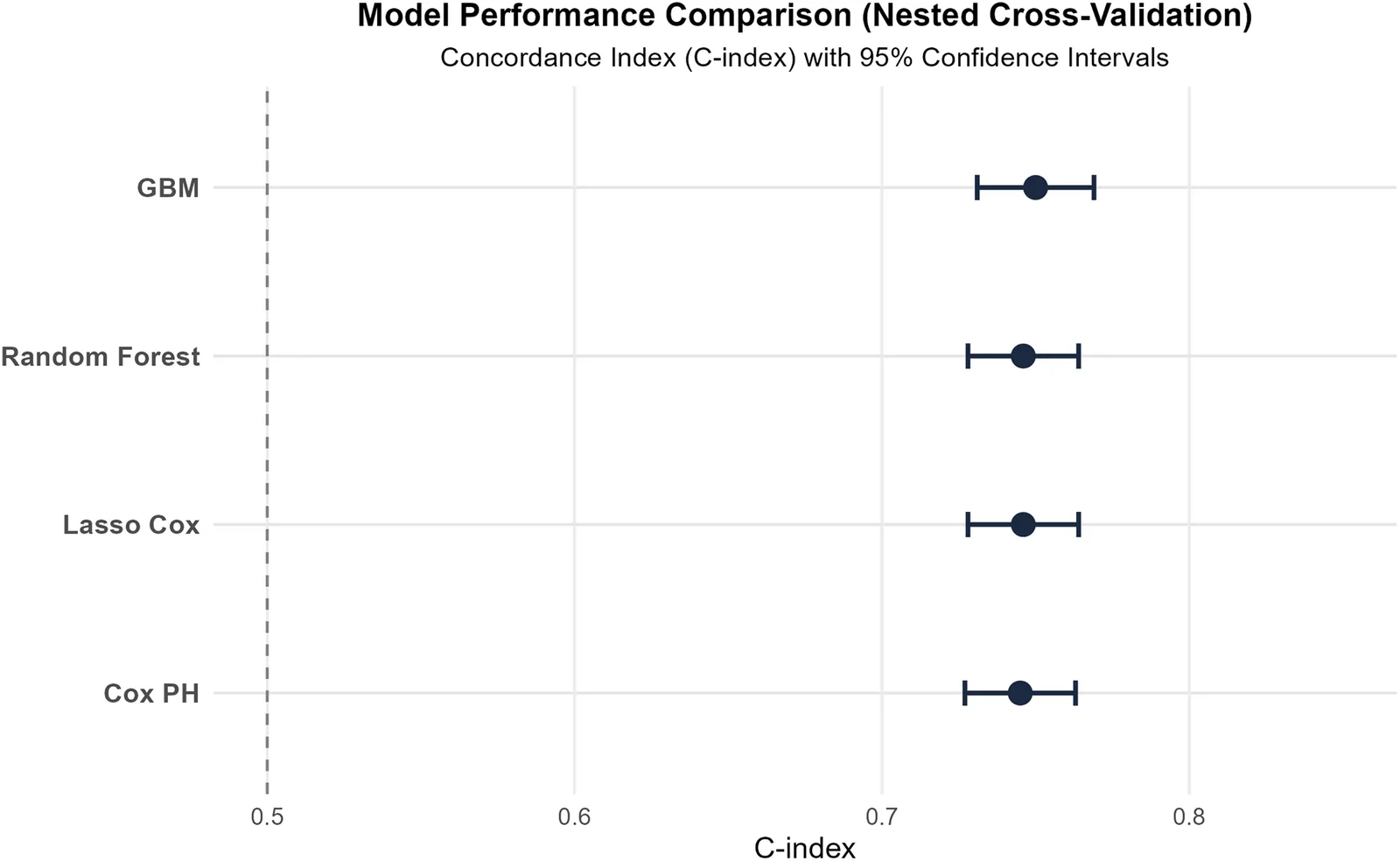
Predictive discrimination of survival models across follow-up time. Concordance index (C-index) with 95% confidence intervals for the four models under nested cross-validation (10-fold mother-level outer folds, 5-fold inner folds for hyperparameter tuning). Under this stricter validation, all four models performed comparably (C-index range: 0.745–0.750), with Gradient Boosting Machine showing a slight advantage. The dashed line indicates chance-level discrimination (C-index = 0.5).

### Predictor Importance and Risk Stratification

Both RSF and GBM identified maternal age and birth interval as the two most important predictors, followed by previous child deaths (Table 5, Fig 3). The machine learning models captured non-linear relationships; for instance, the mortality risk associated with maternal age showed a U-shaped pattern not detected in the Cox PH model (S6 Fig and S7 Fig).

**Table 5:** Variable importance rankings from machine learning models.

Predictor	Random survival forest		Gradient boosting	
	Importance*	Rank	Importance*	Rank
Birth interval	100.0	1	99.1	2
Maternal age	96.3	2	100.0	1
Previous child deaths	78.8	3	87.8	3
Region	6.1	6	17.3	4
Maternal education	15.8	5	11.1	5
Birth order	18.2	4	8.5	6
Wealth index	3.9	7	6.5	7
Child sex	1.2	8	5.2	8
Residence (urban/rural)	0.1	9	1.8	9

*Importance scaled to 100 for the top predictor in each model. RSF = Random Survival Forest; GBM = Gradient Boosting Machine.

**Fig 3:**
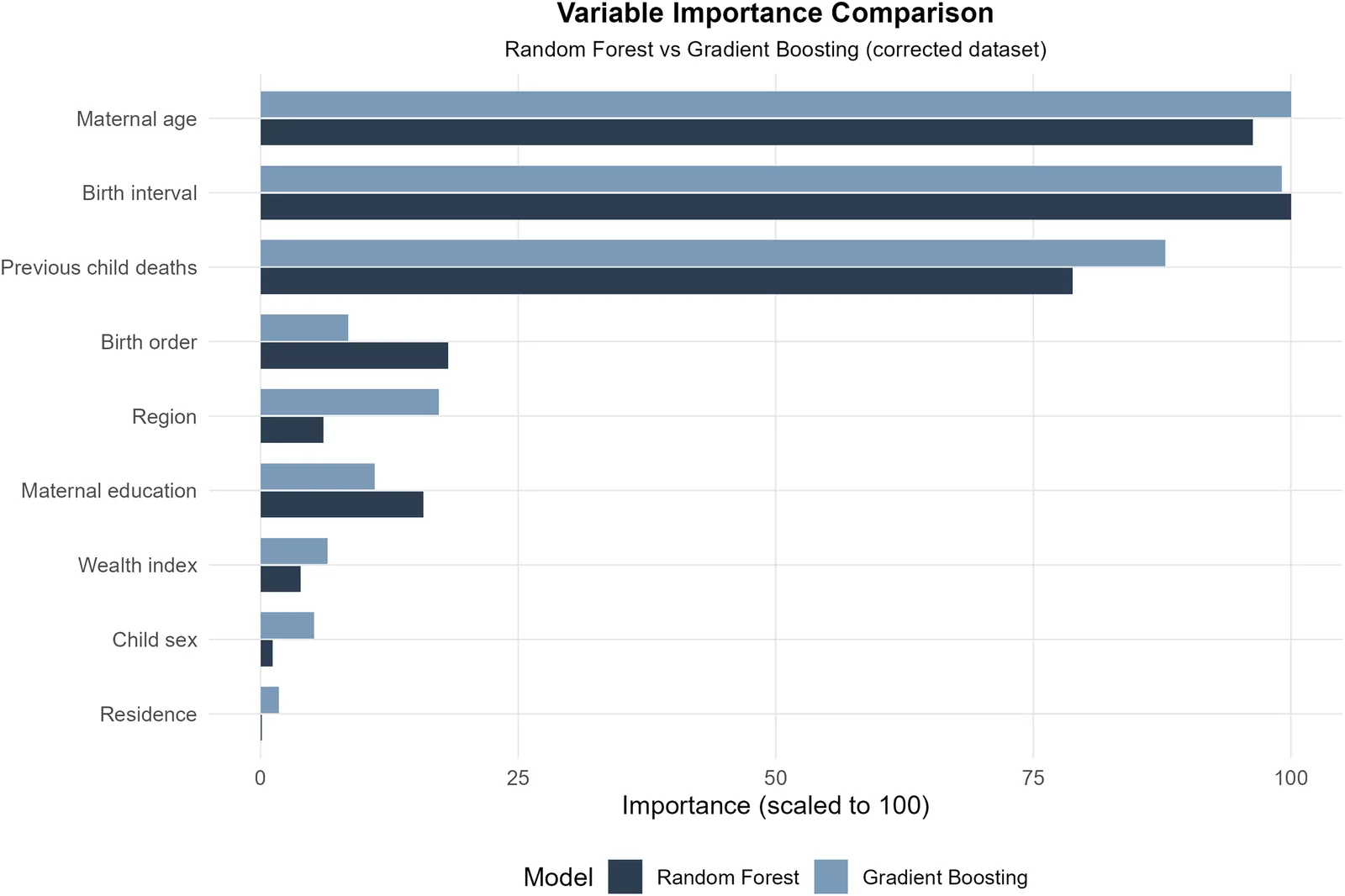
Variable importance comparison of Random Forest (RF) and Gradient Boosting Machine (GBM) models. Both machine learning methods identified birth interval and maternal age as the most important predictors of child mortality, followed by previous child deaths. Socioeconomic factors (wealth index, maternal education, residence) showed relatively lower importance. Values are scaled to 100 for the top predictor in each model.

Variable importance measures from RSF and GBM consistently confirmed maternal age and birth interval as the leading predictors. The Lasso Cox model selected 21 non-zero coefficients, creating a sparse and interpretable predictive model.

Statistical comparisons using DeLong’s test (S7 Table) revealed no statistically significant pairwise differences between models under nested cross-validation. The mean difference in AUC between GBM and Cox PH was 0.000 (95% CI: −0.016 to 0.016; *p* = 0.997), and between GBM and RSF was 0.005 (95% CI: −0.013 to 0.023; *p* = 0.547). Similarly, RSF, Lasso Cox, and Cox PH were statistically indistinguishable (all *p* > 0.6). These results indicate that, under the stricter nested cross-validation protocol, all four models provided comparable predictive performance for under-five child mortality (Fig 4).

**Fig 4:**
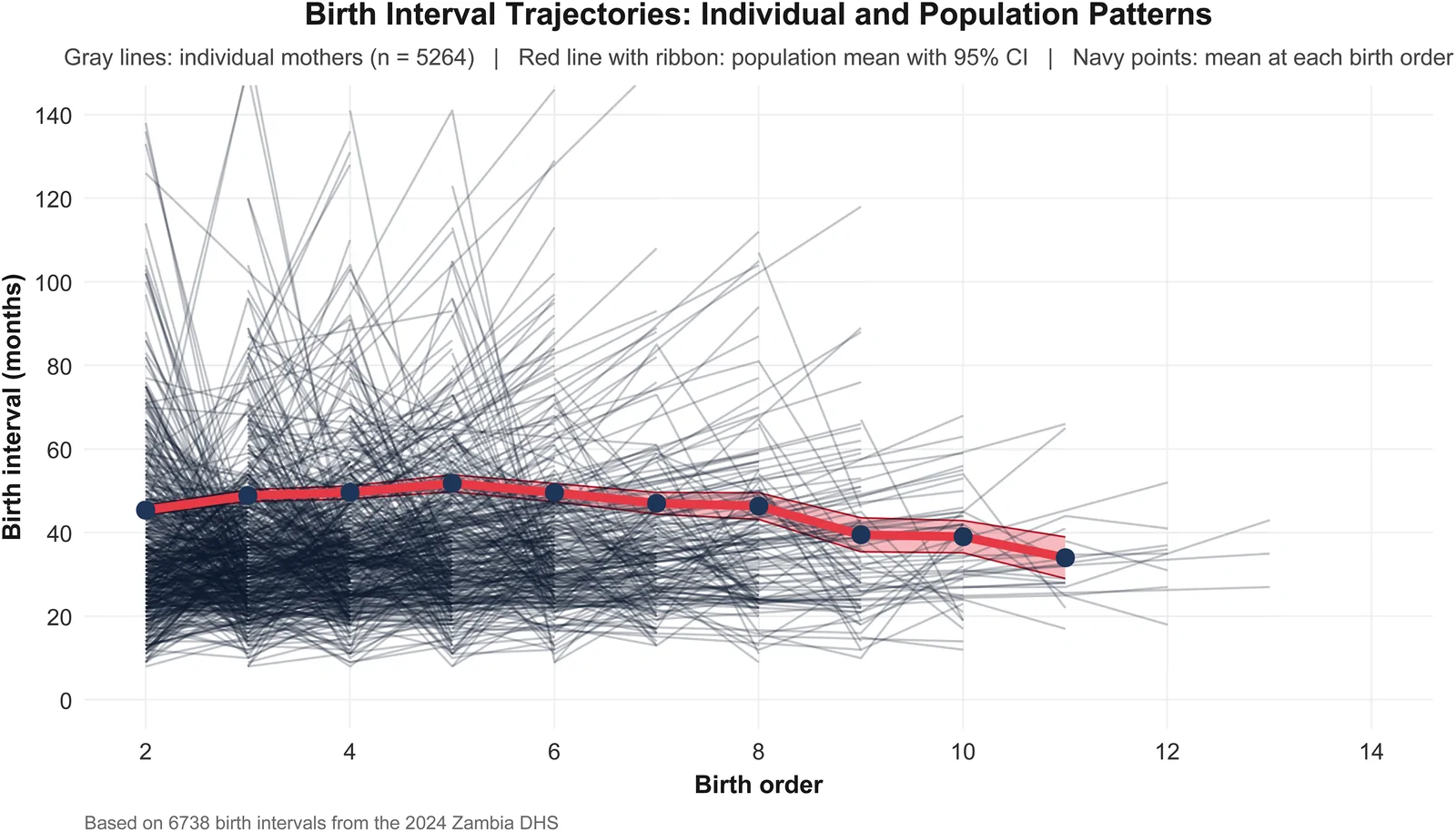
Birth interval trajectories showing both individual maternal patterns and population trends. Gray-blue lines represent individual birth interval trajectories for all 5,264 mothers with multiple births. The solid blue-black line shows the population mean birth interval with a shaded 95% confidence interval, and gold points indicate mean values at each birth order. Birth intervals generally decreased with increasing birth order, from approximately 45.4 months at birth order 2 to 39.0 months at birth order 10, demonstrating substantial between-mother heterogeneity in birth spacing patterns.

### Model calibration

Model calibration was assessed using IPCW-adjusted Brier scores at 12, 24, 36, and 48 months (S5 Fig, S6 Table). All four models showed nearly identical calibration throughout follow-up, with Integrated Brier Scores (IBS) ranging from 0.095 to 0.096 (Cox PH = 0.095; Lasso Cox = 0.095; GBM = 0.095; RSF = 0.096). Brier scores at each time point differed by less than 0.003 between any two models, and all confidence intervals overlapped substantially, indicating no statistically significant difference in calibration. The analysis was truncated at 48 months because no subjects remained at risk at 60 months (numbers at risk at 12, 24, 36, and 48 months: 1,564, 1,126, 751, and 377, respectively). These findings indicate that the four models are equivalently well-calibrated for under-five mortality risk prediction.

## Discussion

The primary finding of this study is that, under nested cross-validation, machine learning models and Cox regression provide comparable predictive performance for under-five child mortality in Zambia. The C-index estimates ranged from 0.745 (Cox PH) to 0.750 (Gradient Boosting Machine), with no statistically significant pairwise differences. This finding emphasizes the critical importance of nested validation when comparing predictive survival models, as non-nested procedures can produce optimistic estimates that may not reflect true out-of-sample performance [4].

### Predictive performance and the role of nested validation

Under nested cross-validation with mother-level outer folds and inner hyperparameter tuning, all four models performed comparably. The mean C-index across folds was 0.745 for Cox PH, 0.746 for Lasso Cox, 0.746 for Random Survival Forest (RSF), and 0.750 for Gradient Boosting Machine (GBM). The small differences did not reach statistical significance in DeLong’s tests. This convergence of performance has important implications. First, it suggests that the complexity of tree-based and ensemble methods does not translate into improved discrimination or calibration for this specific outcome in the Zambian context when evaluated under strict fold separation. Second, it reinforces the value of simpler, more interpretable models such as Cox proportional hazards for risk stratification when performance is equivalent. Third, it underscores that model selection should consider not only predictive accuracy but also interpretability, computational cost, and ease of implementation in resource limited settings [16]. Fourth, the equivalence in calibration (IBS range: 0.095–0.096) indicates that the additional flexibility of machine learning models did not improve the reliability of predicted risks.

Our finding of equivalent performance between ML and Cox models is consistent with a growing body of evidence. Van der Ploeg et al. (2014) showed through simulation studies that modern modeling techniques such as random forests and neural networks require substantially larger effective sample sizes to achieve stable performance and low optimism compared with classical regression models [17]. A recent systematic review of clinical prediction models similarly concluded that machine learning approaches rarely outperform logistic or Cox regression when evaluated on the same data with rigorous validation [18]. Our results extend this pattern to child mortality in a low-resource setting, where the sample size and event rate may limit the ability of tree-based methods to exploit non-linear structure. This convergence suggests that the enthusiasm for ML in survival analysis should be tempered by empirical evidence: in many applied settings, classical models remain competitive.

The nested validation design warrants explicit emphasis. In non-nested designs, hyperparameter tuning and performance estimation are conducted using the same cross-validation procedure, which can introduce optimism into the performance estimates for tree-based models. Cox PH, having no tunable hyperparameters, is less affected by this bias, which can produce an artificially wider gap between ML and Cox models. The nested protocol eliminates this bias and provides more defensible estimates of model performance.

### Variable importance: Birth interval and maternal age lead

The variable importance analysis identified birth interval and maternal age as the two most influential predictors of child mortality, followed by previous child deaths. Both Random Forest and GBM assigned the highest importance scores to birth interval (RSF: 100.0; GBM: 99.1) and maternal age (RSF: 96.3; GBM: 100.0). Previous child deaths remained a strong predictor (RSF: 78.8; GBM: 87.8).

This shift has substantive implications. Birth interval is a modifiable risk factor directly amenable to family planning interventions, and its emergence as the leading predictor reinforces the maternal depletion hypothesis and the importance of optimal birth spacing [5]. Maternal age reflects both biological and social vulnerability particularly adolescent pregnancy and advanced maternal age and its prominence supports targeted interventions for at-risk mothers. Previous child deaths, while still important, may function as a marker for unmeasured familial or environmental risk rather than a direct actionable target.

Our finding that maternal age is protective (HR = 0.909 per year) is consistent with the well-documented U-shaped relationship between maternal age and child mortality. A 2018 analysis of DHS data from 28 sub-Saharan African countries found that mortality was highest for firstborn children with very young mothers, while children of high birth order also experienced elevated mortality risk [19]. A recent survival machine-learning study in low-sociodemographic-index states of India identified maternal age as a key predictor of under-five mortality, with children born to mothers at the extremes of the reproductive age range facing elevated risk [20]. Our variable importance analysis captured this non-linear pattern in the ML models, while the Cox model constrained by linearity reported only the average linear effect. This illustrates a substantive advantage of ML methods: they can identify risk-factor shapes that linear models miss, even when overall discrimination is equivalent.

Socioeconomic factors including wealth index, maternal education, and residence showed comparatively lower importance. The hierarchy is consistent with the descriptive mortality patterns observed (Table 1) and with the Cox model results showing that maternal education and wealth had moderate associations with mortality.

### Joint modeling and birth interval dynamics

The Joint Model (JM) analysis confirmed a statistically significant association between the latent birth interval process and the instantaneous hazard of child mortality (α=0.035, 95% CI: 0.023–0.048, *p* < 0.001). The positive association indicates that shorter birth intervals are associated with increased mortality hazard, supporting the maternal depletion hypothesis. This finding is consistent with the broader literature on birth spacing and child survival [5] and reinforces the public health importance of family planning services.

Our association parameter (α=0.035) is of similar magnitude to birth-interval effects reported in other sub-Saharan African DHS analyses. A 28-year trend analysis in Burkina Faso found that short interbirth intervals (<24 months) were consistently associated with excess mortality, with adjusted hazard ratios ranging from 1.49 to 3.20 across survey rounds [21]. At the multi-country level, a 2018 analysis of DHS data from 28 sub-Saharan African countries confirmed that short preceding birth intervals were associated with above-average child mortality, with the strongest effects observed in the first year of life [19]. Similarly, an analysis of DHS data from eight West African countries found that children born to mothers with birth intervals exceeding two years were significantly less likely to die before age five compared to those with intervals of two years or less [20]. Our joint model, which accounts for the longitudinal birth interval trajectory, provides a statistically efficient estimate consistent with these findings while explicitly addressing informative censoring and measurement error that may bias standard time-varying Cox formulations [14].

Joint modeling has been increasingly applied to child survival research, though most commonly with anthropometric or clinical markers as the longitudinal process rather than birth intervals. A joint modeling study of severe pneumonia in under-five children linked longitudinal respiratory rate trajectories to time-to-recovery, demonstrating that the joint approach provided higher statistical power to detect associations and properly handled outcome-dependent missingness [22]. Our application differs in treating birth interval as the longitudinal process, which has the advantage of being directly modifiable through family planning interventions. This makes the joint-model estimate of α=0.035 more immediately actionable than joint models based on anthropometric markers, which are consequences of underlying health status rather than modifiable exposures.

Individual trajectories showed substantial between-mother heterogeneity in birth spacing patterns (S1 Fig and S5 Fig). The population mean birth interval trajectory declined from 45.4 months at birth order 2 to 39.0 months at birth order 10, with a peak of 51.8 months at birth order 5. This non-linear pattern reflects a complex interaction of reproductive decisions and biological factors, with maternal education modifying the trajectory higher-educated mothers maintained longer intervals across parities.

### Maternal education as a protective factor

Our finding that maternal education is strongly protective (Secondary vs. None: HR = 0.426, 95% CI: 0.318–0.570) is consistent with previous DHS analyses in Zambia and other sub-Saharan African countries. A 2022 study analyzing the 2018 Zambia Demographic and Health Survey found that maternal education had a protective effect for under-five mortality, with higher education associated with reduced hazard of child death [23]. Similarly, a 2025 analysis of the 2018 ZDHS reported that mothers with secondary or higher education had reduced odds of neonatal mortality in rural Zambia [24]. At the multi-country level, a 2016 analysis of DHS data from eight sub-Saharan African countries (including Zambia) demonstrated that under-five mortality rates among children born to mothers without formal education were higher than among children of educated mothers, with children of mothers having 0–6 years of education being about 20% more likely to survive to age five compared to children of mothers with no schooling [25]. Cost-effectiveness modeling of maternal and child health interventions in South Africa has similarly shown that scaling up education-related and health-system interventions could substantially reduce child mortality and accelerate progress toward child survival targets [13]. These findings collectively support the universal importance of maternal education as a fundamental determinant of child survival, operating through multiple pathways including improved health literacy, greater healthcare utilization, and delayed childbearing.

### Time-varying effects and stratified analysis

The proportional hazards assumption was assessed using Schoenfeld residuals. The global PH test showed a significant violation ($\chi^{2}=48.51$, df = 22, *p* = 0.0009), primarily driven by maternal education (*p* = 0.036) and previous child deaths (*p* = 0.052). These findings suggest that the effects of these variables on mortality risk change over the child’s life course — an important consideration for both interpretation and implementation.

To address the PH violation for previous child deaths, we conducted a stratified Cox model, which allows the baseline hazard to differ across categories (no previous deaths, one previous death, two or more) (S4 Table). The stratified model demonstrated consistent effects for other predictors and a C-index of 0.715 (SE = 0.012). The stratified analysis confirmed the protective effects of maternal education and the elevated risk associated with higher birth order.

The PH violation for maternal education is especially interesting. It suggests that the protective effect of education may be strongest in the early months of life and attenuate over time. This could reflect the differential impact of education on early-life behaviors (breastfeeding, vaccination, healthcare seeking) versus later childhood exposures.

### Wealth and child mortality

Descriptive analysis revealed an expected inverse gradient between household wealth and child mortality: mortality was highest in the poorest quintile (13.6%) and lowest in the richest quintile (9.9%) (Table 1). However, in the multivariable Cox model adjusted for maternal, child, and reproductive factors, this gradient was attenuated and no statistically significant association between wealth quintile and child mortality remained after adjustment for birth interval, maternal education, and previous child deaths (all *p* > 0.10; Table 2). The apparent reversal of the wealth gradient observed in some adjusted models is likely attributable to confounding by correlated maternal and reproductive factors — particularly maternal education and birth interval — rather than to a true causal effect of wealth on child survival. These findings suggest that in the Zambian context, the socioeconomic gradient in child mortality operates substantially through maternal education and reproductive behaviors rather than through household wealth per se. This interpretation is consistent with the broader literature, in which maternal education is consistently identified as the dominant socioeconomic determinant of child survival in sub-Saharan Africa, mediating access to healthcare, health literacy, and reproductive decision-making. Given the cross-sectional design, reverse causality cannot be excluded — households that have experienced child deaths may experience subsequent economic decline — and further longitudinal research is needed to disentangle these pathways.

### Comparison with prior Zambian studies

Our findings are consistent with prior analyses of Zambian DHS data. Mulenga et al. (2022), using the 2018 ZDHS, identified maternal education, birth interval, and maternal age as key determinants of under-five survival [23], echoing the hierarchy we observed. Shumba et al. (2025) similarly found that maternal education was protective against neonatal mortality in rural Zambia [24]. Our study extends this body of work in three ways. First, we provide a formal predictive comparison between classical and machine learning survival models under nested cross-validation, an evaluation strategy not previously applied to Zambian under-five mortality data. Second, we apply a joint modeling framework that explicitly accounts for the longitudinal birth interval process, providing a statistically efficient estimate of the maternal depletion effect. Third, we assess calibration in addition to discrimination, an often-overlooked dimension of model performance. Together, these extensions position our findings as both a replication and a methodological advancement of prior Zambian child survival research.

### Multiple births sensitivity analysis

The sensitivity analysis including multiple births (n = 1,053 additional observations) showed that multiple births had significantly higher mortality (HR = 2.50, 95% CI: 2.02–3.10, *p* < 0.001) compared to singletons. This is consistent with the well-documented elevated risk associated with multiple pregnancies, driven by preterm birth, low birth weight, and increased neonatal complications. The decision to exclude multiple births from the primary analysis was justified by the need to maintain the independence assumption in Cox models, where multiple births to the same mother would violate the independence of observations. The sensitivity analysis confirms that including multiples would not change the primary conclusions but does provide important information about this high-risk subgroup.

Future studies should investigate risk factors for mortality specifically among multiple births, ideally using pooled data across multiple DHS surveys or specialized cohort studies with sufficient statistical power to examine this vulnerable population.

### Interpretability and clinical utility

To enhance the practical utility of our findings, we extracted interpretable decision rules from the GBM model using the rpart package. These rules provide healthcare workers with simple, actionable guidance. For example: “If a mother has experienced a previous child death AND the birth interval is less than 24 months AND she has no secondary education, the child’s mortality risk is approximately 3.2 times higher than baseline.” Such rules can be integrated into community health worker protocols without requiring computational resources.

The comparable performance of Cox PH and machine learning models has important implications for implementation. In resource-limited settings where computing infrastructure and data science expertise may be scarce, traditional Cox models with interpretable hazard ratios can provide equivalent predictive performance while being easier to deploy, explain, and maintain. This supports the use of simpler models in many public health applications, reserving more complex methods for settings where they demonstrably improve outcomes.

### Comparison with global literature

Globally, decades of child survival research have established a hierarchy of risk factors where distal socioeconomic determinants — primarily maternal education and household wealth — are considered fundamental protective factors, mediating access to healthcare, sanitation, and nutrition [16]. Intermediate, biological determinants such as optimal birth spacing are also universally recognized as critical modifiable risks [5].

Our results align with this consensus: maternal education was strongly protective, birth interval was the leading predictor, and maternal age was a key determinant. The emergence of birth interval as the top predictor is particularly notable and underscores the potential of family planning interventions to improve child survival in Zambia.

The relatively lower importance of wealth may reflect the convergence of health outcomes across socioeconomic groups in Zambia, or the fact that the DHS wealth index captures long-term asset ownership rather than short-term purchasing power relevant to child health. Alternatively, it may reflect the strong correlation between wealth and other predictors (e.g., education, residence) that reduces its marginal contribution in the multivariable model.

### Study limitations

Our study is subject to several limitations inherent to the use of cross-sectional Demographic and Health Survey data. First, potential recall bias regarding the exact timing of births and deaths may affect both birth interval estimation and survival outcome measurement. Second, the cross-sectional design prohibits definitive causal inference; the identified associations are prognostic and correlational. Third, the use of retrospective birth histories provides a pseudo-longitudinal structure rather than true repeated measurements, which may not fully capture the dynamic relationship between birth intervals and child survival.

Fourth, our validation strategy used nested cross-validation with mother-level outer folds, but survey sampling weights were not incorporated into the machine learning models because standard implementations do not directly support complex survey weights. This may affect the generalizability of our ML-based risk predictions to the target population, although the Cox models did incorporate survey weights and clustering. Future work should explore weighted ensemble methods that can incorporate survey design information.

Fifth, despite our large sample size, the number of events per fold in the nested cross-validation was modest (125 per fold), which limits the statistical power for detecting small differences between models. This may partially explain why no significant differences were detected between models; larger samples or pooled multi-country analyses may reveal meaningful differences.

Sixth, the 60-month time point could not be evaluated for model calibration because no subjects remained at risk at that time point, which truncates our Brier score analysis at 48 months.

Seventh, external validation using independent datasets is essential before implementation. Our models were developed and validated within the 2024 Zambia DHS, but their performance in other contexts — different countries, time periods, or healthcare systems — remains unknown. We recommend prospective validation studies with the Zambia Ministry of Health and testing in other sub-Saharan African countries.

## Conclusion

This study provides a comprehensive comparative analysis of classical and advanced survival modeling techniques for predicting under-five mortality. By leveraging the Zambia Demographic and Health Survey (DHS), we demonstrated that machine learning models, specifically Random Survival Forests (RSF) and Gradient Boosting Machines (GBM), provide predictive accuracy and discrimination comparable to the Cox Proportional Hazards framework. Under nested cross-validation, all four models performed equivalently, and the machine learning models captured non-linear complexities and interactions inherent in socio-economic and maternal health data, offering additional interpretive value for identifying high-risk children even when overall discrimination was equivalent.

A key contribution of this research is the application of a Joint Model, which successfully integrated longitudinal birth interval markers with survival outcomes. The significant association parameter confirmed the “maternal depletion hypothesis,” showing that the fluctuation of birth intervals is a time-varying predictor of child survival that is often oversimplified in classical models.

Furthermore, the variable importance analysis identified birth interval and maternal age as the most influential predictors of mortality, followed by previous child deaths. This suggests that a mother’s obstetric history is a proxy for underlying household vulnerabilities and biological risks. From a policy perspective, our findings advocate for a shift toward data-driven, precision public health strategies. Interventions in Zambia should prioritize mothers with a history of child loss and focus on integrated family planning services to optimize birth spacing.

In conclusion, integrating joint modeling and machine learning into public health surveillance offers a meaningful advancement in our ability to understand and predict child mortality. Given the equivalent performance of simpler and more complex models, public health implementation should prioritize interpretable, scalable solutions, while future research should explore the deployment of these models into real-time clinical decision-support systems to assist front line healthcare workers in targeted resource allocation.

## Supporting information

S1 Fig Complete individual birth interval trajectories (spaghetti plot). All individual maternal birth interval trajectories for mothers with multiple births (n = 5,264 mothers, 6,738 birth intervals). Each blue-black line represents one mother’s birth intervals across successive births, showing substantial between-mother heterogeneity in birth spacing patterns. Birth orders are plotted from 2 to 14, as first births have no preceding interval. The plot demonstrates wide variation in reproductive timing strategies, with some mothers maintaining consistent spacing while others show irregular patterns or trends across parities.
(EPS)

S2 Fig Population mean birth interval profile with confidence intervals. Mean birth interval trajectory for the entire population with 95% confidence intervals (shaded area). Birth intervals show a non-linear pattern across birth orders, starting at 45.4 months for birth order 2, peaking at 51.8 months for birth order 5, and gradually decreasing to 39.0 months by birth order 10. Error bars at each birth order represent 95% confidence intervals around the mean. The plot is based on 6,738 birth intervals from the 2024 Zambia DHS.
(EPS)

S3 Fig Birth interval profiles stratified by maternal education level. Mean birth interval trajectories for four education categories: no education (dark blue-black), primary (medium blue-black), secondary (light blue-black), and higher education (palest blue-black). At birth order 2, the education gradient was: no education (40.5 months), primary (39.7 months), secondary (50.6 months), and higher education (58.4 months). Mothers with secondary or higher education maintained substantially longer birth intervals across all birth orders, while the difference between no education and primary was minimal. These patterns suggest that maternal education beyond the primary level is associated with optimal birth spacing practices.
(EPS)

S4 FigKaplan-Meier survival curves stratified by birth interval category.Survival probability estimates for six birth interval groups: short intervals (<18 months, darkest blue-black), moderately short intervals (18–23 months, dark blue-black), adequate intervals (24–35 months, medium blue-black), long intervals (36–47 months, light blue-black), very long intervals (≥48 months, palest blue-black), and first birth (primary blue-black). The log-rank test revealed statistically significant differences between categories ($\chi^{2}=399$, df = 5, *p* < 0.0001). Children born following very short birth intervals (<18 months) exhibited the lowest survival probability (~60% at 60 months), whereas those born after very long intervals (≥48 months) demonstrated the highest survival probability (~91% at 60 months). This strong dose-response relationship supports the maternal depletion hypothesis.(EPS)

S5 Fig Calibration plots and Brier scores for the four survival models (Cox PH, Lasso Cox, Random Forest, and GBM) at 12, 24, 36, and 48 months. Brier scores were computed on the held-out test set using inverse probability of censoring weighting (IPCW) as implemented in the riskRegression R package. Shaded bands represent 95% confidence intervals. Lower Brier scores indicate better calibration. The plot is truncated at 48 months because no subjects remained at risk at 60 months (numbers at risk at 12, 24, 36, and 48 months: 1,564, 1,126, 751, and 377, respectively). All four models demonstrated nearly identical calibration throughout follow-up, with Integrated Brier Scores (IBS) ranging from 0.095 to 0.096 (Cox PH = 0.095; Lasso Cox = 0.095; GBM = 0.095; RSF = 0.096). No statistically significant differences in calibration were observed at any time point.
(EPS)

S6 Fig SHAP (SHapley Additive exPlanations) summary plot for the Gradient Boosting Machine (GBM) model, showing the contribution of each predictor to the model’s predictions. The x-axis represents the mean SHAP value (impact on predicted mortality risk), and the y-axis lists the features in descending order of importance. Maternal age (29.7) and birth interval (29.4) emerged as the two most influential predictors, followed by previous child deaths (26.0). Socioeconomic factors (region, maternal education, wealth index) and child characteristics (sex, residence) showed relatively smaller contributions.
(EPS)

S7 Fig Partial dependence plots (PDPs) for the four most important predictors in the Gradient Boosting Machine (GBM) model: maternal age, birth interval, previous child deaths, and birth order. Each plot shows the marginal effect of a single predictor on the predicted mortality risk, holding all other predictors constant at their mean values. The plots demonstrate a U-shaped relationship for maternal age (lowest risk at approximately 30 years), a decreasing relationship for birth interval (shorter intervals associated with higher risk), an increasing relationship for previous child deaths, and an increasing relationship for birth order. These patterns support the maternal depletion hypothesis and highlight the importance of reproductive timing and obstetric history in child survival.
(EPS)

S1 Table Comparison of complete-case and multiple imputation results. Comparison of Cox proportional hazards coefficients from complete-case analysis versus multiple imputation using the MICE algorithm (10 imputed datasets). Coefficients are on the log-hazard scale. The imputation model included all predictors and the survival outcome. Differences between complete-case and imputed estimates were small in magnitude and did not alter the direction or significance of any association. Structural missingness in the birth interval variable (first births with no preceding interval) was not imputed, as these values are undefined rather than missing at random.
(PDF)

S2 TableProportional hazards assumption test results.Results of the Schoenfeld residual-based test of the proportional hazards assumption for each covariate in the multivariable Cox model. The global test was statistically significant ($\chi^{2}=48.51$, df = 22, *p* = 0.0009), indicating an overall violation of the proportional hazards assumption, primarily driven by maternal education (*p* = 0.036) and previous child deaths (*p* = 0.052). All other covariates satisfied the assumption (all *p* > 0.05). To address the violation for previous child deaths, a stratified Cox model was fitted using previous child deaths category as the stratification variable (see Table S4). PH, proportional hazards.(PDF)

S3 Table Variance Inflation Factors (VIF) for predictors. Variance Inflation Factors for all predictors included in the multivariable Cox model, used to assess multicollinearity. All VIF values were below 5 (range: 1.01–4.03), indicating no problematic multicollinearity. The highest VIF values were observed for birth order (4.03) and maternal age (3.98), which is expected given their natural correlation with parity and reproductive age. Values below the conventional threshold of 10 (and the more conservative threshold of 5) confirm that multicollinearity does not materially affect the stability of the estimated coefficients. VIF, Variance Inflation Factor.
(PDF)

S4 Table Stratified Cox model by previous child deaths category. Results of a stratified Cox proportional hazards model in which the baseline hazard was allowed to vary across strata defined by previous child deaths category (no previous deaths, one previous death, two or more previous deaths). This approach addresses the violation of the proportional hazards assumption detected for previous child deaths (Table S2). Effects for all other covariates are estimated as common across strata. Model fit: C-index = 0.715 (SE = 0.012); likelihood ratio test = 660.4 (df = 21, *p* < 0.001). HR, hazard ratio; CI, confidence interval.
(PDF)

S5 TableHyperparameter tuning details (nested cross-validation).Hyperparameter ranges evaluated and final selected values for each machine learning model. Optimization was performed using inner 5-fold cross-validation nested within an outer 10-fold mother-level cross-validation loop. For the Lasso Cox model, the regularization parameter λ was selected by inner cross-validation; the final value ($\lambda_{\min}=0.015$) minimized the mean cross-validated partial likelihood deviance. RSF, Random Survival Forest; GBM, Gradient Boosting Machine; CV, cross-validation.(PDF)

S6 Table Brier scores and Integrated Brier Scores (IBS) by model and time point. Brier scores were computed on the held-out test set using inverse probability of censoring weighting (IPCW) as implemented in the riskRegression R package. Lower Brier scores indicate better calibration. Brier scores are reported at 12, 24, 36, and 48 months. The table is truncated at 48 months because no subjects remained at risk at 60 months (numbers at risk at 12, 24, 36, and 48 months: 1,564, 1,126, 751, and 377, respectively), rendering 60-month estimates unstable. IBS = Integrated Brier Score over 12–48 months (trapezoidal integration). All four models performed equivalently (IBS range: 0.095–0.096), with no statistically significant differences at any time point. IPCW, inverse probability of censoring weighting; IBS, Integrated Brier Score.
(PDF)

S7 Table DeLong’s test for pairwise model comparisons. Pairwise comparisons of time-dependent AUC at 24 months between all four survival models, using DeLong’s test for correlated ROC curves with bootstrap resampling (500 iterations). Difference = difference in AUC between the two models; a positive value indicates the first-listed model has higher AUC. All *p*-values exceed 0.05, indicating no statistically significant difference in discrimination between any pair of models. GBM, Gradient Boosting Machine; RSF, Random Survival Forest; AUC, area under the receiver operating characteristic curve; CI, confidence interval; ns, not significant.
(PDF)
