# Supplementary material for: Predicting under-five child mortality in Zambia: Machine learning versus parametric survival models
Source: PLoS One. 2026 Oct 22;21(10):e0361197. doi: 10.1371/journal.pone.0361197 (PMC13690213; doi:10.1371/journal.pone.0361197)
Supplement: Supplementary file 1 [file pone.0361197.s008.pdf]

Table S1. Comparison of Complete-Case and Multiple Imputation Results

**Variable**	**Complete Case**	**Imputed**	**Difference**
mother_age	−0.0952	−0.0799	0.0153
child_sexFemale	−0.2969	−0.2828	0.0141
mother_eduPrimary	−0.1764	−0.3339	−0.1575
mother_eduSecondary	−0.8543	−1.1716	−0.3173
mother_eduHigher	−0.9178	−1.2410	−0.3232
wealth_indexPoorer	0.2033	0.2191	0.0158
wealth_indexMiddle	0.3406	0.3436	0.0030
wealth_indexRicher	0.5865	0.6087	0.0222
wealth_indexRichest	0.7800	0.8252	0.0452
residenceRural	0.0133	0.0123	−0.0010
region2	−0.0490	−0.1425	−0.0935
region3	0.0040	−0.1725	−0.1765
region4	−0.0074	−0.0227	−0.0153
region5	−0.0132	−0.0268	−0.0136
region6	−0.4493	−0.4271	0.0222
region7	−0.0759	−0.0507	0.0252
region8	−0.5364	−0.5914	−0.0550
region9	−0.2449	−0.3494	−0.1045
region10	0.2910	0.2078	−0.0832
birth_order	0.0142	−0.0093	−0.0235
prev_deaths	0.4996	0.5538	0.0542
birth_interval	−0.0237	−0.0222	0.0015

Note: Comparison of coefficients from Cox PH models using complete-case analysis versus multiple imputation (10 imputed datasets). Coefficients are on the log-hazard scale.
