# Supplementary material for: Predicting under-five child mortality in Zambia: Machine learning versus parametric survival models
Source: PLoS One. 2026 Oct 22;21(10):e0361197. doi: 10.1371/journal.pone.0361197 (PMC13690213; doi:10.1371/journal.pone.0361197)
Supplement: Supplementary file 2 [file pone.0361197.s009.pdf]

Table S2. Proportional Hazards Assumption Test Results

Variable	Chisq	df	p-value
mother_age	0.279	1	0.597
child_sex	1.728	1	0.189
mother_edu	8.565	3	0.036
wealth_index	5.362	4	0.252
residence	0.333	1	0.564
region	16.259	9	0.062
birth_order	0.868	1	0.352
prev_deaths	3.780	1	0.052
birth_interval	2.442	1	0.118
**GLOBAL**	**48.514**	**22**	**0.0009**

Note: Global test $p = 0.0009$ indicates PH violation, primarily driven by maternal education ($p = 0.036$) and previous child deaths ($p = 0.052$).
