# Supplementary material for: Predicting under-five child mortality in Zambia: Machine learning versus parametric survival models
Source: PLoS One. 2026 Oct 22;21(10):e0361197. doi: 10.1371/journal.pone.0361197 (PMC13690213; doi:10.1371/journal.pone.0361197)
Supplement: Supplementary file 3 [file pone.0361197.s010.pdf]

Table S3. Variance Inflation Factors (VIF) for Predictors

Variable	VIF	Interpretation
birth_order	4.030	Acceptable
mother_age	3.978	Acceptable
wealth_indexRichest	2.466	Acceptable
mother_eduSecondary	2.331	Acceptable
wealth_indexRicher	2.309	Acceptable
mother_eduPrimary	2.059	Acceptable
residenceRural	1.958	Acceptable
wealth_indexMiddle	1.794	Acceptable
region10	1.701	Acceptable
region3	1.718	Acceptable
region5	1.744	Acceptable
region2	1.567	Acceptable
region7	1.634	Acceptable
wealth_indexPoorer	1.599	Acceptable
region9	1.585	Acceptable
region6	1.351	Acceptable
region8	1.425	Acceptable
prev_deaths	1.256	Acceptable
birth_interval	1.248	Acceptable
mother_eduHigher	1.444	Acceptable
child_sexFemale	1.011	Acceptable

Note: All VIF values < 5 indicate no problematic multicollinearity.
