# Supplementary material for: Predicting under-five child mortality in Zambia: Machine learning versus parametric survival models
Source: PLoS One. 2026 Oct 22;21(10):e0361197. doi: 10.1371/journal.pone.0361197 (PMC13690213; doi:10.1371/journal.pone.0361197)
Supplement: Supplementary file 4 [file pone.0361197.s011.pdf]

Table S4. Stratified Cox Model by Previous Child Deaths Category

Variable	HR	95% CI	p-value
mother_age	0.923	0.902–0.944	< 0.001
child_sexFemale	0.744	0.649–0.851	< 0.001
mother_eduPrimary	0.847	0.695–1.033	0.101
mother_eduSecondary	0.463	0.359–0.598	< 0.001
mother_eduHigher	0.418	0.241–0.727	0.002
wealth_indexPoorer	1.207	1.000–1.457	0.050
wealth_indexMiddle	1.315	1.055–1.639	0.015
wealth_indexRicher	1.771	1.338–2.345	< 0.001
wealth_indexRichest	2.129	1.468–3.088	< 0.001
residenceRural	1.030	0.840–1.262	0.779
birth_order	0.955	0.887–1.028	0.218
birth_interval	0.977	0.972–0.981	< 0.001

Model Fit: C-index = 0.715 (SE = 0.012); LR test = 660.4 (df = 21, $p < 0.001$).

Note: Stratified by previous child deaths category.
