# Supplementary material for: Predicting under-five child mortality in Zambia: Machine learning versus parametric survival models
Source: PLoS One. 2026 Oct 22;21(10):e0361197. doi: 10.1371/journal.pone.0361197 (PMC13690213; doi:10.1371/journal.pone.0361197)
Supplement: Supplementary file 5 [file pone.0361197.s012.pdf]

Table S5. Hyperparameter Tuning Details (Nested Cross-Validation)

Model	Parameter	Range	Final Value
Random Survival Forest	ntree	100, 200, 500	500
	mtry	$\sqrt{p}$, $p/3$, $p/2$	$\sqrt{p}$
	nodesize	5, 10, 15, 20	15
	splitrule	logrank, C	logrank
Gradient Boosting Machine	n.trees	500, 1000, 2000	1000
	interaction.depth	2, 3, 4	3
	shrinkage	0.01, 0.05, 0.1	0.01
	n.minobsinnode	10, 15, 20	10
Lasso Cox	lambda	10^{-4} to 10^{1}	$\lambda_{\text{min}} = 0.015$

Note: Optimization used inner 5-fold cross-validation nested within an outer 10-fold mother-level loop.
