# Supplementary material for: Predicting under-five child mortality in Zambia: Machine learning versus parametric survival models
Source: PLoS One. 2026 Oct 22;21(10):e0361197. doi: 10.1371/journal.pone.0361197 (PMC13690213; doi:10.1371/journal.pone.0361197)
Supplement: Supplementary file 6 [file pone.0361197.s013.pdf]

Table S6. Brier Scores and Integrated Brier Scores (IBS) by Model and Time Point

Model	12 months	24 months	36 months	48 months	IBS
Cox PH	0.056	0.090	0.105	0.124	0.095
Lasso Cox	0.056	0.090	0.105	0.124	0.095
Random Forest	0.056	0.091	0.107	0.125	0.096
GBM	0.056	0.090	0.104	0.124	0.095

Note: Brier scores were computed on the held-out test set using inverse probability of censoring weighting (IPCW) as implemented in `riskRegression::Score()`. Lower scores indicate better calibration. The table is truncated at 48 months because no subjects remained at risk at 60 months (numbers at risk at 12, 24, 36, and 48 months: 1,564, 1,126, 751, and 377, respectively). IBS = Integrated Brier Score over 12–48 months. All four models performed equivalently (IBS range: 0.095–0.096).
