# Supplementary material for: Predicting under-five child mortality in Zambia: Machine learning versus parametric survival models
Source: PLoS One. 2026 Oct 22;21(10):e0361197. doi: 10.1371/journal.pone.0361197 (PMC13690213; doi:10.1371/journal.pone.0361197)
Supplement: Supplementary file 7 [file pone.0361197.s014.pdf]

Table S7. DeLong's Test for Pairwise Model Comparisons

**Comparison**	**Difference**	**95% CI**	**Z-stat**	**p-value**
GBM vs Cox PH	0.0000	$(-0.016, 0.016)$	0.004	0.997
GBM vs Lasso Cox	-0.0003	$(-0.016, 0.015)$	-0.031	0.975
GBM vs RSF	0.0053	$(-0.013, 0.023)$	0.603	0.547
RSF vs Cox PH	-0.0057	$(-0.029, 0.018)$	-0.462	0.644
RSF vs Lasso Cox	-0.0058	$(-0.028, 0.016)$	-0.500	0.617
Lasso Cox vs Cox PH	0.0005	$(-0.002, 0.003)$	0.449	0.653

Note: DeLong's test for time-dependent AUC at 24 months using bootstrap resampling (500 iterations). All p-values > 0.05 indicate no statistically significant difference between any pair of models. GBM = Gradient Boosting Machine; RSF = Random Survival Forest.
