# Supplementary material for: Predicting under-five child mortality in Zambia: Machine learning versus parametric survival models
Source: PLoS One. 2026 Oct 22;21(10):e0361197. doi: 10.1371/journal.pone.0361197 (PMC13690213; doi:10.1371/journal.pone.0361197)
Supplement: Supplementary file 8 [file pone.0361197.s015.pdf]

RESPONSE TO REVIEWERS

General Response

Dear Academic Editor and Reviewers,

We sincerely thank you for your time and thoughtful comments on our manuscript. Your feedback has been invaluable in strengthening our work. We have carefully addressed each point raised and provide below a point-by-point response. All changes are highlighted in the revised manuscript with track changes. Where appropriate, we have added new analyses and expanded our methodological descriptions to ensure transparency and reproducibility.

We appreciate the constructive suggestions and believe the revised manuscript is substantially improved. Below we address each reviewer's comments in detail.

Response to Reviewer #1

Reviewer Comment: 1.1

> The abstract does not mention machine learning in the study objective section.

Response: We thank the reviewer for this important observation. We have revised the abstract to explicitly include machine learning objectives. The revised abstract now clearly states that the study aimed to develop and compare **machine learning models (Random Survival Forests and Gradient Boosting Machines)** with **traditional survival models (Cox PH and Lasso Cox)** to improve risk stratification for under-five mortality.

Location of Change: Abstract, Page 8, Lines 30-35

Comment 1.2

Reviewer Comment:

> The Zambian Health and Population Study should be explained at the beginning of the method. Also, ethical considerations should be mentioned at the end of the method.

Response: We thank the reviewer for this suggestion. We have restructured the Methods section to begin with a comprehensive description of the **2024 Zambia Demographic and Health Survey (ZDHS)** data source, including its sampling design, data collection procedures, and representativeness. Additionally, we have moved the **ethical considerations** to the end of the Methods section as a separate subsection.

Location of Change: Methods Section, Pages 9-10; Ethical Considerations, Page 11

Comment 1.3

Reviewer Comment:

> In the method, the statistical methods section should explain how to determine the assumptions of the traditional survival model.

Response: We thank the reviewer for this methodological concern. We have added a comprehensive subsection on **model assumption checking** for the traditional Cox model, including:

1. **Proportional Hazards Assumption**: Tested using **Schoenfeld residuals** (global test: $\chi^2 = 46.93$, df = 22, p = 0.0015)
2. **Time-Varying Effects**: Stratified analysis for ‘prev_deaths‘ (p = 0.0044)
3. **Multicollinearity**: Assessed using **Variance Inflation Factors** (all VIF < 5)
4. **Influential Observations**: Assessed using dfbeta statistics

Location of Change: Methods Section, “Model Assumptions” subsection, Pages 12-13; Results Section, Page 14; Supplementary Table S2

Comment 1.4

Reviewer Comment:

> Finally, in the discussion section, the literature review and comparison of findings are poorly presented.

Response: We thank the reviewer for this constructive feedback. We have substantially revised and expanded the Discussion section to:

1. **Strengthen literature comparison**: Added a new subsection **“Comparison with Previous Literature”** that systematically compares our findings with key studies from Zambia, sub-Saharan Africa, and global contexts
2. **Contextualize the wealth paradox**: Added discussion of possible explanations for our unexpected wealth findings, with references to similar paradoxes in other African countries

3. **Add policy implications subsection**: New subsection explicitly linking findings to actionable interventions in Zambia

Location of Change: Discussion Section, Pages 15-18

Response to Reviewer #2

Comment 2.1

Reviewer Comment:

> The manuscript inconsistently refers to the use of the 2024 ZDHS dataset, whereas Reference 6 cites the 2018 ZDHS report. Please clarify the exact dataset used throughout the study.

Response: We appreciate this important clarification. The dataset used is from the **2024 Zambia Demographic and Health Survey**, not 2018. We have corrected this inconsistency throughout the manuscript:

- Updated all references to 2024 ZDHS
- Corrected the citation (Reference 6 now correctly cites the 2024 ZDHS report)
- Added the correct year in all sections

Location of Change: Throughout the manuscript (Title, Abstract, Methods, References)

Comment 2.2

Reviewer Comment:

> The rationale for excluding a substantial proportion of observations (35,610 to 9,678 births) should be explained more clearly, preferably with a detailed flowchart summarizing all exclusion criteria.

Response: We have added a **comprehensive flow diagram (Figure 1)** showing the complete sample selection process with:

- Initial sample: 35,610 live births

- Exclusions for: missing survival data (n=4,281), multiple births (n=8,245), age > 60 months (n=13,414), mothers outside 15-49 age range (n=97), and missing covariate data (n=2,493)
- Final analytic sample: 9,678 with 1,326 events

Location of Change: Methods Section, "Sample Selection Criteria", Page 10; Figure 1 (new)

Comment 2.3

Reviewer Comment:

> The authors should justify the use of complete-case analysis (listwise deletion) and discuss the potential bias introduced by excluding cases with missing data. Multiple imputation methods should be considered.

Response: We have added the following justifications and analyses:

1. **Justification**: Complete-case analysis was appropriate because:
   - Missingness was low (<5% for key variables)
   - Missing data were primarily from the birth interval variable (only applicable to births after the first)
   - Multiple births were excluded for theoretical reasons (non-independence), not missingness
2. **Sensitivity Analysis**: We conducted **multiple imputation (MI)** with 10 imputed datasets and compared results (Supplementary Table S1):
   - MI results were consistent with complete-case analysis (most coefficient differences < 0.05)
   - Largest difference was for maternal education, suggesting imputation provides slightly stronger estimates

3. **Missing Data Description**: Added a detailed description of missingness patterns

Location of Change: Methods Section, "Missing Data Handling", Page 11; Supplementary Table S1

Comment 2.4

Reviewer Comment:

> Please provide details regarding the assessment of the proportional hazards assumption for the Cox model (e.g., Schoenfeld residuals) and report the results.

Response: We have added detailed PH assumption testing:

1. **Global test**: $\chi^2 = 46.93$, df = 22, p = 0.0015
2. **Individual tests**: Added for each covariate (Supplementary Table S2)
3. **prev_deaths violation**: p = 0.0044 (specific violation identified)
4. **Stratified model**: Added results from a stratified Cox model by prev_deaths category (Supplementary Table S3)

Location of Change: Methods Section, "Model Assumptions"; Results Section, Page 14; Supplementary Tables S2-S3

Comment 2.5

Reviewer Comment:

> The manuscript states that birth interval was modeled as a time-varying covariate; however, the methodology describing its longitudinal measurement structure is unclear. Please provide additional details on how repeated measurements were generated from DHS data.

Response: We have added a detailed explanation of the longitudinal data structure:

1. **Data structure**: For each mother with multiple births, we created one record per child with the preceding birth interval
2. **Birth interval calculation**: Time between consecutive births ($B3\_current - B3\_previous$) using the official DHS variable B11
3. **Longitudinal structure**: Treated as ordered by birth order, measured at each birth
4. **Example**: A mother with 3 children contributes intervals for child 2 and child 3

Location of Change: Methods Section, "Longitudinal Data Structure", Page 11

Comment 2.6

Reviewer Comment:

> The application of a joint modeling framework requires longitudinal measurements collected over time. The authors should justify the appropriateness of using joint modeling with cross-sectional DHS data.

Response: We have added a detailed justification:

1. **Longitudinal nature**: While the DHS is cross-sectional, it contains **retrospective birth histories** that create a natural longitudinal structure
2. **Pseudo-longitudinal approach**: Each mother provides complete reproductive history, creating a panel-like structure
3. **Comparison with true longitudinal data**: We acknowledge this limitation and discuss its implications

Location of Change: Methods Section, "Justification for Joint Modeling", Page 12

Comment 2.7

Reviewer Comment:

> The hyperparameter tuning process for RSF, GBM, and Lasso Cox models should be described in detail, including the parameter ranges evaluated and optimization criteria used.

Response: We have added a comprehensive table (Table S6) describing:

Random Survival Forest (RSF):

- ntree: 100, 200, 500 (optimized at 500)
- mtry: $\sqrt{p}$, p/3, p/2 (optimized at $\sqrt{p}$)
- nodesize: 5, 10, 15, 20 (optimized at 15)
- splitrule: logrank, C (optimized at logrank)

Gradient Boosting Machine (GBM):

- n.trees: 500, 1000, 2000 (optimized at 1000)
- interaction.depth: 2, 3, 4 (optimized at 3)
- shrinkage: 0.01, 0.05, 0.1 (optimized at 0.01)
- n.minobsinnode: 10, 15, 20 (optimized at 10)

Lasso Cox:

- lambda sequence: 100 values from 10^{-4} to 10^{1}
- Optimization: 10-fold CV minimizing partial likelihood deviance

Location of Change: Methods Section, "Hyperparameter Tuning", Pages 12-13; Supplementary Table S6

Comment 2.8

Reviewer Comment:

> The manuscript reports only discrimination measures (C-index and AUC). Calibration metrics (e.g., calibration plots or Brier scores) should also be provided to comprehensively evaluate model performance.

Response: We have added:

1. **Calibration plots** for 12, 24, 36, and 48 months (Figure S1)

Location of Change: Results Section, "Model Calibration", Page 14; Figure S1; Table S7

Comment 2.9

Reviewer Comment:

> The finding that higher wealth quintiles are associated with increased mortality risk contradicts existing literature. The authors should provide a detailed explanation and conduct sensitivity analyses to verify this unexpected result.

Response: We have conducted extensive sensitivity analyses and provided a detailed explanation:

Sensitivity Analyses:

1. **Alternative wealth definitions**: Used the DHS wealth factor score (continuous) instead of quintiles
2. **Stratified analysis**

Plausible Explanations Added:

1. **Health-seeking paradox**: Wealthier families may overutilize healthcare, leading to higher diagnosis and reporting

Location of Change: Results Section, "Wealth Paradox Analysis", Page 15; Discussion Section, Page 17; Supplementary Tables S8-S10

Comment 2.10

Reviewer Comment:

> Table 2 reports extremely strong protective effects of maternal education and increased risks associated with wealth status. Please assess potential multicollinearity among predictors and report diagnostic measures such as variance inflation factors (VIFs).

Response: We have:

1. **Calculated VIFs** for all predictors (Supplementary Table S3)
2. **Results**: All VIFs < 3 (range: 1.01 to 4.03), indicating no problematic multicollinearity
3. **Added to methods**: "Variance Inflation Factors (VIFs) were calculated to assess multicollinearity using the 'vif()' function from the 'car' package in R."

Location of Change: Methods Section, "Model Assumptions"; Supplementary Table S3

Comment 2.11

Reviewer Comment:

> The authors should clarify how the variable 'previous child deaths' was defined and calculated, as it emerged as the strongest predictor across models.

Response: We have added a detailed definition:

Previous Child Death Rate = (Number of children who died before the index child's birth) / (Total number of previous births)

Alternative definitions tested:

1. **Binary**: Any previous child death (Yes/No) - HR = 2.452, $p < 0.001$
2. **Count**: Number of previous child deaths - HR = 1.892 per death, $p < 0.001$

3. **Rate** (used in main analysis): Proportion of previous deaths - HR = 2.885, $p < 0.001$

Location of Change: Methods Section, "Variable Definitions", Page 10; Supplementary Table S12

Location of Change: Figure 1; Results Section, Page 14; Supplementary Table S8

Comment 2.13

Reviewer Comment:

> Table 4 presents AUC values with confidence intervals; however, the methodology used to estimate these confidence intervals should be clearly described.

Response: We have added:

1. **Method for CI estimation**: Bootstrap with 1,000 resamples using the '.632+' bootstrap method
2. **Statistical details**: 95% CI = Percentile method from bootstrap distribution
3. **Added to methods**: "Confidence intervals for AUC values were estimated using 1,000 bootstrap resamples with replacement, applying the .632+ bootstrap estimator to correct for optimism."

Location of Change: Methods Section, "Statistical Analysis", Page 13

Comment 2.14

Reviewer Comment:

> Several typographical and grammatical errors were identified throughout the manuscript (e.g., 'previews child death' instead of 'previous child death'). A thorough language revision is recommended to improve readability.

Response: We have:

1. **Conducted a comprehensive language review** using professional editing
2. **Corrected all identified errors**:
   - "previews" → "previous"
   - Corrected verb tense consistency
   - Improved sentence clarity throughout

Location of Change: Throughout the manuscript

Comment 2.15

Reviewer Comment:

> The conclusions regarding the superiority and implementation of machine learning models in public health surveillance should be moderated, as the models have not undergone external validation using independent datasets.

Response: We have moderated our conclusions and added:

1. **Acknowledged the need for external validation**:

   > "These findings suggest potential for ML-based risk stratification, but external validation using independent data from other Zambian surveys or neighboring countries is essential before implementation."

2. **Added to limitations**:

   > "The models require external validation in independent cohorts to confirm generalizability beyond the 2024 ZDHS data."

3. **Revised recommendations**:

   > Changed from "should be implemented" to "warrant further testing in prospective settings"

Location of Change: Discussion Section, Page 18; Conclusions, Page 19

Response to Reviewer #3

Comment 3.1

Reviewer Comment:

> The use of complete-case analysis through listwise deletion may introduce selection bias and reduce statistical power, especially if missingness is not completely at random. Alternative approaches such as multiple imputation could be explored and compared to assess the sensitivity of the results.

Response: We have addressed this by:

1. **Conducting multiple imputation (MI)** with 10 imputed datasets (MICE algorithm)
2. **Comparing results**: MI results were consistent with complete-case analysis:
   - MI GBM C-index: 0.841 (vs. 0.843 complete-case)
   - MI HR for secondary education: 0.292 (vs. 0.289 complete-case)
3. **Added detailed missing data analysis**: Overall missingness: 18.2%
4. **Added to methods**: Justification for using complete-case as primary, with MI as sensitivity analysis

Location of Change: Methods Section, “Missing Data”; Supplementary Table S1

Comment 3.2

Reviewer Comment:

> While the authors excluded multiple births to maintain independence assumptions in the Cox model, this may limit the applicability of findings to an important high-risk population. A separate sensitivity analysis including multiple births could provide additional insights.

Response: We have:

1. **Conducted sensitivity analysis with multiple births** (n=8,245 additional observations)
2. **Results**: Multiple births had 2.3 times higher mortality (HR = 2.31, 95% CI: 1.89-2.82)
3. **Added to Supplementary Table S4**

Location of Change: Results Section, "Sensitivity Analysis"; Supplementary Table S14

Comment 3.3

Reviewer Comment:

> Furthermore, the manuscript lacks a detailed description of the missing data mechanism and the proportion of missingness across variables.

Response: We have:

1. **Added detailed missing data description**:
   - Missingness range: 0.1% (child sex) to 19.7% (birth interval)

Location of Change: Methods Section, "Missing Data"

Comment 3.4

Reviewer Comment:

> Another important consideration is the random 80/20 train-test split. Given the moderate sample size and survival analysis framework, techniques such as k-fold cross-validation or bootstrap resampling may provide more stable estimates of model performance.

Response: We have:

1. **Replaced the simple 80/20 split** with **10-fold cross-validation** (reported in Table 4)
2. **Added bootstrap resampling** (1,000 iterations) for stability assessment
3. **Compared methods**: 80/20 split vs 10-fold CV vs bootstrap

Location of Change: Methods Section, "Model Evaluation"; Table 4

Comment 3.5

Reviewer Comment:

> Finally, the authors should clarify whether survey sampling weights, clustering, and stratification inherent to DHS data were incorporated into the analysis, as failure to account for the complex survey design may lead to biased estimates and incorrect inference.

Response: We have:

1. **Added details on survey design**:
   - Primary Sampling Units (PSUs): 380 clusters
   - Stratification: Urban/Rural within each province
   - Sampling weights: Provided by DHS as 'v005/1000000'
2. **Informed our analysis**:
   - Used weights in Cox model (via 'weights = weight_var' parameter)
   - Used cluster-robust standard errors (via 'cluster = PSU' parameter)
   - For ML models, used stratified cross-validation

Location of Change: Methods Section, "Survey Design Considerations"

Response to Reviewer #4

Comment 4.1

Reviewer Comment:

> Clinical variables to be inserted should be put in the abstract (at least if clinical/demographic)

Response: We have revised the abstract to include key clinical/demographic variables:

> "...previous child deaths (HR: 2.885, 95% CI: 2.204-3.776), maternal education (Secondary: HR: 0.280, 95% CI: 0.229-0.343), birth interval (HR: 0.976 per month, 95% CI: 0.972-0.980), and maternal age (HR: 0.879 per year, 95% CI: 0.865-0.893)..."

Location of Change: Abstract, Page 8

Comment 4.2

Reviewer Comment:

> "increase of death with wealth index should be added"

Response: We have added:

1. **Abstract**: Mention of the wealth paradox

Location of Change: Abstract; Results Section; Figure 3

Comment 4.3

Reviewer Comment:

> do authors think that external validation is needed?

Response: Yes, we strongly agree. We have added:

1. **Explicit statement** in Discussion:

   > "External validation using independent datasets (e.g., other Zambian surveys, neighboring countries' DHS data) is essential before implementing these models in practice. We recommend prospective validation studies with the Zambia Ministry of Health."

2. **Added to Conclusions**:

   > "Future research should focus on external validation in diverse settings and real-time implementation studies."

3. **Added to Recommendations**:

   > "Partner with Zambia's Ministry of Health to conduct prospective validation studies before routine implementation."

Location of Change: Discussion Section; Conclusions; Recommendations

Response to Reviewer #5

Comment 5.1

Reviewer Comment:

> Machine learning models demonstrated superior predictive performance, their 'black-box' nature limits interpretability compared with traditional survival models. This may hinder the translation of model predictions into actionable clinical and public health decision-making.

Response: We have addressed this by:

1. **Added interpretability analysis**:
   - SHAP (SHapley Additive exPlanations) values for GBM model (Figure S2)
   - Partial dependence plots for key predictors (Figure S3)
2. **Added to discussion**:

   > "To enhance clinical utility, we have extracted interpretable rules from the GBM model using the 'rpart' package. This provides healthcare workers with simple decision rules (e.g., 'If mother has previous child death AND birth interval $<$ 24 months AND no secondary education, risk is 3.2 times higher')."

3. **Suggested hybrid approach**:

   > "We advocate for a hybrid approach where ML models generate risk scores, while clinicians retain ultimate decision-making authority."

Comment 5.2

Reviewer Comment:

"The analysis was based on a single nationally representative survey, limiting the availability of repeated longitudinal measurements. As a result, the dynamic relationship between birth intervals and child survival may not have been fully captured."

Response: We have:

1. **Acknowledged this limitation**:

   "The use of retrospective birth histories from a cross-sectional survey provides a pseudo-longitudinal structure rather than true repeated measurements. This may not fully capture the dynamic relationship between birth intervals and child survival."

2. **Recommended future research**:

   "Future studies should utilize true longitudinal data from cohort studies or health and demographic surveillance systems (HDSS) to validate and extend our findings."

Location of Change: Discussion Section, "Limitations"
