# Supplementary material for: Predicting under-five child mortality in Zambia: Machine learning versus parametric survival models
Source: PLoS One. 2026 Oct 22;21(10):e0361197. doi: 10.1371/journal.pone.0361197 (PMC13690213; doi:10.1371/journal.pone.0361197)
Supplement: Supplementary file 10 [file pone.0361197.s017.pdf]

Response to Reviewers

Manuscript ID: PONE-D-26-00277R3

Title: *Predicting Under-Five Child Mortality in Zambia: Machine Learning Versus Parametric Survival Models*

Kassaye Getaneh Arge
Department of Statistics, Samara University, Ethiopia
kassayegetaneh76@gmail.com

General Response

Dear Dr. José Antonio Ortega,

We sincerely thank you for the opportunity to revise our manuscript and for your careful review. We appreciate your confirmation that the revision meets the reviewers' requirements, and we are grateful for the detailed editorial checks that identified three remaining minor issues in R2. We have addressed all three points in this R3 revision.

We also thank the reviewers for their constructive feedback throughout the revision process. We acknowledge that Reviewer #3 and Reviewer #5 recommended acceptance, and we have focused our revisions on the concerns raised by the Academic Editor, Reviewer #1, and Reviewer #4.

All changes in the revised manuscript are highlighted using `latexdiff` in the file *Revised Manuscript with Track Changes*.

Summary of major changes across all revisions:

- Corrected the multiple-birth variable from `M19` to `B0`.
- Rebuilt the sample-selection flowchart so that all exclusion numbers are mathematically consistent with the final analytic sample of 9,362 singleton births and 1,236 mortality events (13.2%).

- Clarified the validation strategy: nested 10-fold cross-validation with mother-level outer folds and inner 5-fold hyperparameter tuning; a single 80/20 split was used only as a sensitivity analysis.
- Clarified the handling of missing data and the structural absence of birth-interval data for first births.
- Expanded the description of the joint-modeling approach.
- Clarified how within-mother correlation and survey design were handled.
- Added discussion of discrepancies between ML- and traditional-model predictors.
- Added a limitation regarding the non-incorporation of survey weights in the machine-learning models.
- Added an explicit limitation regarding the estimation strategy and within-mother correlation.
- **(R3)** Updated the system abstract to match the corrected manuscript abstract.
- **(R3)** Corrected the Data Availability Statement from 2018 to 2024 ZDHS and moved it to its proper place in the manuscript.
- **(R3)** Corrected the Table S4 → Table S5 cross-reference.

Response to the Academic Editor (R3)

Editor Comment 1: System Abstract Still Contains Old Results

Editor comment:

> "While the abstract in the main text has been updated, and it shows the correct results, the systems abstract still has the old results. Make sure to change it."

Response: We thank the Editor for catching this inconsistency. We confirm that the online submission system's abstract field still contained the outdated results from the earlier version. We have now replaced the system abstract with the corrected abstract, which matches the abstract in the manuscript exactly.

Action taken:

- Updated the **Abstract field** on the online submission form in Editorial Manager.
- The corrected abstract now reports the accurate results: C-indices ranging from 0.745 (Cox PH) to 0.750 (GBM), with no statistically significant pairwise differences between models.
- The system abstract is now **identical** to the abstract in the manuscript.

Location of change: Online submission form, Abstract field; Manuscript, Abstract [Page 1].

Editor Comment 2: Data Availability Statement Still Refers to DHS 2018

Editor comment:

> "The data availability statement still shows DHS 2018 instead of DHS 2024. Note that at the end of the manuscript you provide a corrected statement, however this is not included in its proper place."

Response: We apologize for this oversight. We confirm that (1) the online submission form's Data Availability Statement field still referred to the 2018 ZDHS, and (2) the corrected statement appeared at the end of the manuscript rather than in its proper place.

Action taken:

- Updated the **Data Availability Statement field** on the online submission form to refer to the **2024 Zambia Demographic and Health Survey (ZDHS)**.
- Moved the corrected Data Availability Statement to its **proper place** in the manuscript, immediately after the Acknowledgments and before the References.
- Removed the duplicate statement from the end of the manuscript.

Corrected Data Availability Statement (now in its proper place):

> "The datasets analyzed in this study were obtained from The DHS Program (`https://dhsprogram.com/`) following a formal application and approval for the research project titled *Predicting Under-Five Child Mortality in Zambia: Machine Learning Versus Parametric Survival Models*. The authors are authorized by The DHS Program to use the **2024 Zambia Demographic and Health**

> **Survey (ZDHS)** data strictly for statistical reporting and analysis. In accordance with the data use agreement, all data were treated as confidential, and no attempts were made to identify individual respondents or households. As the redistribution of DHS micro-level data is prohibited, the data are not provided within the manuscript or supporting information; however, interested researchers may apply for access directly through The DHS Program portal at `https://dhsprogram.com/data/available-datasets.cfm`."

Location of change: Online submission form, Data Availability Statement field; Manuscript, new *Data Availability Statement* section placed after Acknowledgments and before References.

Editor Comment 3: Incorrect Table Numbering

Editor comment:

> "(Line 301): The text states that hyperparameter configurations were selected based on Table S4. However, Table S4 presents the Stratified Cox model results, while hyperparameter configurations are presented in Table S5."

Response: We thank the Editor for identifying this incorrect cross-reference. We confirm that the hyperparameter configurations are presented in **Table S5**, not Table S4.

Action taken:

- Corrected the cross-reference in the *Machine Learning Model Performance* section from Table S4 to **Table S5**.

Corrected sentence:

> "The final ensemble models reached optimal convergence and stability, with hyperparameter configurations selected based on the nested cross-validated performance metrics illustrated in **Table S5**."

Location of change: Manuscript, *Machine Learning Model Performance* section [Page 12].

Response to the Academic Editor (R1/R2)

Editor Comment 1: Remaining Inconsistencies

Editor comment:

> "These reviewers point that inconsistencies still exist in the manuscript regarding the sample selection and the treatment of missing data, the validation strategy and the description of the data round. Please address these inconsistencies."

Response: We thank the Academic Editor for this summary. We have systematically audited the manuscript and corrected all remaining inconsistencies:

- **Sample selection:** The sample-selection flowchart (Figure 1) and the accompanying text have been rebuilt so that all exclusion numbers are mathematically consistent. The corrected flow is: 35,610 initial live births → 1,053 multiple births excluded → 25,139 children aged > 60 months excluded → 56 mothers outside 15–49 years excluded → 0 missing key predictors → **9,362 singleton births with 1,236 deaths (13.2%)**.
- **Treatment of missing data:** We have clarified that birth interval (B11) is structurally missing for first births, that structural missingness was not imputed, and that multiple imputation was used only as a sensitivity analysis for the remaining, non-structural missingness.
- **Validation strategy:** We have clarified that the primary validation strategy is **nested 10-fold cross-validation with mother-level outer folds and inner 5-fold hyperparameter tuning**, and that a single 80/20 train–test split was used only as a sensitivity analysis.
- **Description of the data round:** We have confirmed that the dataset used is the **2024 Zambia Demographic and Health Survey (ZDHS)** and have corrected all references to the data round throughout the manuscript and the Data Availability Statement.

Location of change: Methods, *Sample Selection Criteria* [Page 3]; Methods, *Model Evaluation* [Page 6]; Methods, *Joint Modeling Approach* [Page 5–6]; Results [Page 8]; Figure 1 [Page 7]; Data Availability Statement (submission form).

Editor Comment 2: Estimation Strategy and Within-Mother Correlation

Editor comment:

> "They also suggest some limitations regarding the estimation strategy. Some of these aspects could be included as limitations of the analysis (eg: not taking into consideration correlation among births to the same mother would lead to a

biased estimate of uncertainty, and there could be more efficient methods that take this into consideration. However, the validation strategy is not affected by this: you can still compare the performance of different models irrespective of se estimation of coefficients)."

Response: We thank the Academic Editor for this important clarification. We agree that within-mother correlation can affect the estimation of standard errors and that more efficient methods exist. We have addressed this in two ways:

1. **We have clarified how within-mother correlation was handled in each modeling framework:**
   - For Cox PH and Lasso Cox models, we used cluster-robust standard errors with the primary sampling unit (V001) as the cluster variable.
   - For machine-learning models (RSF, GBM), we used mother-level cross-validation, where all births from the same mother were kept in the same fold.
   - The joint model explicitly accounted for within-mother correlation through mother-specific random intercepts and random slopes.
2. **We have added an explicit limitation acknowledging that the estimation strategy may not fully account for within-mother correlation in all models, and that this may affect the precision of uncertainty estimates, although it does not invalidate the model-performance comparison.**

Added limitation text:

"Our estimation strategy has limitations. For the Cox PH and Lasso Cox models, cluster-robust standard errors were used to account for within-mother correlation, but these may not fully capture the dependence structure. For the machine-learning models, mother-level cross-validation was used, but the models themselves do not explicitly model within-mother correlation. More efficient methods, such as mixed-effects survival models or frailty models, could provide more accurate uncertainty estimates. However, we emphasize that the validation strategy—nested mother-level cross-validation—remains valid for comparing the predictive performance of different models, irrespective of the standard-error estimation of coefficients."

Location of change: Methods, *Model Evaluation* [Page 6]; Limitations [Page 18].

Response to Reviewer #1

Comment 1.1: Dataset Consistency (2024 vs. 2018 ZDHS)

Reviewer comment:

> "There is still an inconsistency regarding the use of the 2024 versus 2018 Zambia DHS dataset, particularly in the Data Availability Statement. This should be corrected throughout the manuscript."

Response: We thank the reviewer for catching this. The dataset used in this study is the **2024 Zambia Demographic and Health Survey (ZDHS)**. We have corrected all references throughout the manuscript and the Data Availability Statement so that the year is consistently 2024.

Action taken:

- Updated all occurrences of "2018" to "2024".
- Updated Reference 6 to: *Zambia Statistics Agency (ZamStats) and ICF. Zambia Demographic and Health Survey 2024. DHS Program Final Report (FR397).*
- Revised the Data Availability Statement to explicitly refer to the 2024 ZDHS.

Location of change: Abstract [Page 1]; Methods [Page 3]; Results [Page 8]; Acknowledgments [Page 25]; Reference 6 [Page 26]; Data Availability Statement (submission form).

Comment 1.2: Validation Strategy

Reviewer comment:

> "The manuscript and response letter are not fully consistent regarding the use of the 80/20 train–test split versus 10-fold cross-validation. The final validation strategy should be clearly described."

Response: We apologize for the inconsistency. Our **primary validation strategy was nested 10-fold cross-validation with mother-level outer folds and inner 5-fold hyperparameter tuning**, as reported in Table 4 of the revised manuscript. A single 80/20 train–test split was used only as a sensitivity analysis, and the results were consistent with the nested cross-validation.

Action taken:

- Clarified the primary validation strategy (nested 10-fold cross-validation with mother-level outer folds).
- Clarified that the 80/20 split was used only as a sensitivity analysis.
- Added a clear statement to the Methods section.

Revised text:

> "Model performance was estimated using nested 10-fold cross-validation with mother-level outer folds: all births from the same mother were kept in the same fold to account for within-mother correlation. Hyperparameter tuning was conducted in an inner 5-fold loop within each outer fold. This nested structure prevents information leakage between tuning and evaluation, providing unbiased estimates of model performance. A single 80/20 train–test split was used only as a sensitivity analysis, and results were consistent with the nested cross-validation."

Location of change: Methods, *Model Evaluation* [Page 6]; Results [Page 11]; Table 4 [Page 11].

Comment 1.3: Missing Data and Birth Interval

Reviewer comment:

> "The handling of missing data, particularly the structural absence of birth interval information for first births, requires clearer explanation. The relationship between complete-case analysis and multiple imputation should also be clarified."

Response: We have clarified the missing-data mechanism and the structural nature of missingness in birth interval.

Action taken:

- Added a clear statement that **birth interval (B11) is structurally missing for first births**.
- Clarified that structural missingness was **not imputed**, and first births were retained as a separate structural category where appropriate.
- Clarified the role of multiple imputation (sensitivity analysis for remaining, non-structural missingness).

Revised text:

> "The birth interval variable (B11) is structurally missing for first births, as there is no preceding birth interval to calculate. This accounts for the majority of missingness in this variable. Multiple imputation was conducted as a sensitivity analysis for the remaining missing data, and structural missingness (first births) was not imputed."

Location of change: Methods [Page 6]; Results [Page 8].

Comment 1.4: Joint Modeling Approach

Reviewer comment:

> "The application and structure of the joint model require further methodological clarification, particularly regarding the linkage between maternal birth-interval trajectories and individual child survival outcomes."

Response: We have added a detailed explanation of the joint model structure.
Action taken:

- Described the **longitudinal submodel** (linear mixed-effects model with random intercepts and random slopes for birth order).
- Described the **survival submodel** (Cox proportional hazards with current-value association structure).
- Described the **linkage** via the association parameter α.
- Described **model fitting** using the `JMbayes2` package in R with Bayesian MCMC estimation.

Revised text in Methods:

> "The joint model consists of two linked submodels: a longitudinal submodel for the birth-interval trajectory and a survival submodel for child mortality. The longitudinal submodel was a linear mixed-effects model with random intercepts and slopes for birth order. The survival submodel was a Cox proportional hazards model with the current value of the longitudinal process. The association parameter α quantifies the strength and direction of the relationship. The model was fitted using `JMbayes2` with Bayesian MCMC estimation (12,000 iterations, 2,000 burn-in, thinning of 5)."

Location of change: Methods, *Joint Modeling Approach* [Page 5–6].

Comment 1.5: Modeling and Survey Design

Reviewer comment:

> "The authors should clarify how within-mother correlation, complex survey design, and potential dependence between observations were handled, particularly for the machine-learning models."

Response: We have clarified how within-mother correlation and survey design were handled.

Action taken:

- For Cox models: survey sampling weights (`v005/1000000`) and cluster-robust standard errors (`V001`) were used.
- For ML models: mother-level cross-validation was used.
- We added a limitation acknowledging that ML models do not directly incorporate survey weights.

Revised text:

> "Within-mother correlation was addressed in three ways. First, for Cox PH and Lasso Cox models, we used cluster-robust standard errors with the primary sampling unit (V001) as the cluster variable. Second, for machine learning models (RSF, GBM), we used mother-level cross-validation, where all births from the same mother were kept in the same fold. Third, the joint model explicitly accounted for within-mother correlation through mother-specific random intercepts and random slopes."

Location of change: Methods, *Data Source* and *Model Evaluation* [Page 6]; Limitations [Page 18].

Response to Reviewer #4

We thank the reviewer for the detailed and constructive comments.

Comment 4.1: Abstract – Higher Than Linear Regression

Reviewer comment:

> "Higher than linear regression should be reported also."

Response: We have added an explicit comparison between machine-learning models and the traditional Cox regression model in the Abstract. In the revised analysis under nested cross-validation, the four models performed comparably, and this is now clearly stated.

Revised text:

> "Under nested cross-validation, the four survival models demonstrated comparable predictive performance, with C-indices ranging from 0.745 (Cox PH) to 0.750 (Gradient Boosting Machine) and Integrated Brier Scores ranging from 0.095 to 0.096; pairwise comparisons across models were not statistically significant (DeLong's test: all $p > 0.05$)."

Location of change: Abstract [Page 1].

Comment 4.2: Abstract – Clinical Features

Reviewer comment:

> "At least the most important clinical features for ML analysis should be added."

Response: We have added the most important clinical and demographic features to the Abstract.

Revised text:

> "Variable importance analysis identified birth interval and maternal age as the most influential predictors of child survival, followed by previous child deaths."

Location of change: Abstract [Page 1].

Comment 4.3: Sample-Selection Flowchart Inconsistencies

Reviewer comment:

> "The sample-selection flowchart is mathematically inconsistent. The reported exclusions sum to 28,530, which would leave 7,080 observations, not 9,678. Moreover, the text reports 1,780 exclusions for missing survival information, whereas

the flowchart reports 4,281. The reported 8,245 multiple births is also implausibly high. Most importantly, the flowchart identifies multiple births using M19 ¿ 1, although the DHS multiple-birth variable is B0; M19 concerns birth weight. The entire cohort-construction code must be audited and rerun."

Response: We sincerely thank the reviewer for identifying this critical error. We confirm that `M19` is the DHS variable for birth weight, and `B0` is the correct variable for multiple births. We have:

1. **Corrected the code**: replaced `M19 > 1` with `B0 > 0`. The result with the corrected data has been changed and the conclusion slightly revised based on the new result.
2. **Rebuilt the flowchart** (Figure 1) so that all exclusion numbers are mathematically consistent with the final analytic sample of 9,362 singleton births and 1,236 mortality events (13.2%).
3. **Corrected the text** so that the exclusion counts match the flowchart.

Revised sample-selection numbers:

- Initial live births: 35,610
- Excluded for multiple births (B0 ¿ 0): 1,053
- Excluded for age ¿ 60 months: 25,139
- Excluded for maternal age ¡ 15 or ¿ 49 years: 56
- Excluded for missing key predictors: 0
- Final analytic sample: 9,362 singleton births
- Mortality events: 1,236 (13.2%)

Location of change: Methods, *Sample Selection Criteria* [Page 3]; Figure 1 [Page 7]; Results [Page 8].

Comment 4.4: Model Validation (Nested, Mother-Level CV)

Reviewer comment:

> "Model validation is inadequate and inconsistently described. Hyperparameter tuning and performance estimation appear to use the same cross-validation procedure; nested, mother-level cross-validation is required. Stratified cross-validation does not account for survey weights or household and PSU clustering. The manuscript must also explain whether weights were used in model fitting, performance estimation, calibration, and bootstrap resampling."

Response: We thank the reviewer for this methodological concern. In the revised manuscript, we have implemented **nested 10-fold cross-validation with mother-level outer folds and inner 5-fold hyperparameter tuning**. This ensures that hyperparameter tuning and performance estimation are conducted in separate loops, preventing information leakage and providing unbiased performance estimates. We have also clarified where survey weights were used.

Action taken:

1. Clarified the nested validation strategy in the Methods section.
2. Confirmed that all cross-validation folds were constructed at the **mother level**.
3. Clarified where survey weights were used.
4. Added a limitation acknowledging that survey weights were not incorporated into the machine-learning models.

Where survey weights were used:

Stage	Cox PH / Lasso Cox	RSF / GBM
Model fitting	Sampling weights (v005) + cluster-robust SEs (V001)	Weights not supported
Performance estimation	Nested 10-fold mother-level CV	Nested 10-fold mother-level CV
Calibration	Brier scores on held-out folds	Brier scores on held-out folds
Bootstrap	1,000 iterations at mother level	1,000 iterations at mother level

Added limitation text:

"Our validation strategy used nested 10-fold cross-validation with mother-level outer folds to obtain unbiased performance estimates. However, survey sampling weights were not incorporated into the machine learning models, as standard implementations do not directly support complex survey weights. This may affect the generalizability of our ML-based risk predictions to the target population, although the Cox models did incorporate survey weights and clustering. Future work should explore weighted ensemble methods that can incorporate survey design information."

Location of change: Methods, *Model Evaluation* [Page 6]; Limitations [Page 18].

Comment 4.5: Variable Selection

Reviewer comment:

"Why did authors choose variables by themselves and not exploiting models?"

Response: Predictors were selected based on established literature. To assess whether model-based variable selection would yield different results, we fitted a Lasso Cox model, which performs automatic variable selection. The Lasso model selected 21 non-zero coefficients, consistent with the primary analysis.

Location of change: Methods, *Variables* [Page 4]; Results, *Machine Learning Model Performance* [Page 12].

Comment 4.6: Discrepancies Between ML and Traditional Statistics

Reviewer comment:

"Discrepancies between predictors from ML and traditional statistics should be added."

Response: We have added a discussion of discrepancies between ML and traditional statistical models.

Added text:

"The variable importance rankings from the machine-learning models differed from the statistical-significance rankings in the Cox PH model. While birth interval and maternal age were the most important predictors in both approaches,

ML models prioritized maternal age and birth interval more highly than the Cox model, which emphasized maternal education and previous child deaths. This difference reflects the distinct objectives of the two approaches: traditional statistics focus on significant associations, while ML prioritizes predictive accuracy."

Location of change: Results, *Variable Importance Rankings from Machine Learning Models* [Page 12]; Discussion [Page 14].

Response to Reviewer #3

Reviewer comment:

"All comments have been addressed. I recommend acceptance of the manuscript for publication."

Response: We sincerely thank the reviewer for the positive assessment and for recommending acceptance of our manuscript.

Response to Reviewer #5

Reviewer comment:

"I would like to thank the authors for their valuable work. I recommend accepting the manuscript for publication."

Response: We sincerely thank the reviewer for the kind words and for recommending acceptance of our manuscript.

Additional Editorial Requests

Data Availability Statement

We have updated the Data Availability Statement to reflect the 2024 ZDHS and to provide a clear path for access:

"The datasets analyzed in this study were obtained from The DHS Program (`https://dhsprogram.com/`) following a formal application and approval for the research project titled *Predicting Under-Five Child Mortality in Zambia:*

Machine Learning Versus Parametric Survival Models. The authors are authorized by The DHS Program to use the 2024 Zambia Demographic and Health Survey (ZDHS) data strictly for statistical reporting and analysis. In accordance with the data use agreement, all data were treated as confidential, and no attempts were made to identify individual respondents or households. As the redistribution of DHS micro-level data is prohibited, the data are not provided within the manuscript or supporting information; however, interested researchers may apply for access directly through The DHS Program portal at `https://dhsprogram.com/data/available-datasets.cfm`."

Financial Disclosure

The authors received no specific funding for this work. There were no funders involved in study design, data collection and analysis, decision to publish, or preparation of the manuscript.

Reference List

We have reviewed the reference list and confirm that it is complete, correct, and contains no retracted articles. No changes to the reference list were required.

Summary of Changes by Location

**Location**	**Changes made**
Abstract [Page 1]	Added ML-vs-regression comparison; added clinical features; corrected data year.
Online form, Abstract field	Replaced outdated abstract with corrected abstract matching the manuscript.
Online form, Data Availability field	Updated from 2018 ZDHS to 2024 ZDHS.
Methods, Data Source [Page 3]	Clarified 2024 ZDHS; clarified survey design; clarified weights.
Methods, Sample Selection [Page 3]	Corrected multiple-birth variable (`B0`); corrected exclusion counts.
Methods, Variables [Page 4]	Clarified previous-child-death definition; clarified B11.

Methods, Model Evaluation [Page 6]	Clarified nested 10-fold CV; clarified missing-data handling; added limitation on survey weights in ML models.
Methods, Joint Modeling [Page 5–6]	Expanded description of submodels and fitting.
Results [Pages 8–13]	Updated numbers; added discussion of ML-vs-traditional discrepancies.
Discussion [Pages 14–18]	Added discussion of ML-vs-traditional discrepancies; added limitations.
Limitations [Page 18]	Added survey weights in ML models; added estimation-strategy limitation.
Manuscript, after Acknowledgments	Added corrected Data Availability Statement in its proper place.
Manuscript, end of document	Removed duplicate Data Availability Statement.
Manuscript, *Machine Learning Model Performance* [Page 12]	Corrected cross-reference from Table S4 to Table S5.
Figure 1 [Page 7]	Rebuilt flowchart with corrected numbers and correct variable.
References [Page 26]	Corrected Reference 6 to 2024 ZDHS.

Closing

We are grateful for the constructive feedback that has substantially improved our manuscript. We hope that the revised version now meets all the requirements of *PLOS ONE*.

Respectfully submitted,

Kassaye Getaneh Arge
Department of Statistics
Samara University
Samara, Ethiopia
Email: kassayegetaneh76@gmail.com
