# Supplementary material for: Predicting under-five child mortality in Zambia: Machine learning versus parametric survival models
Source: PLoS One. 2026 Oct 22;21(10):e0361197. doi: 10.1371/journal.pone.0361197 (PMC13690213; doi:10.1371/journal.pone.0361197)
Supplement: Peer review history

# Peer review: Decision Letter 0

**DOI:** 10.1371/journal.pone.0361197.r001

**Authors:** Guichuan Lai

17 Jun 2026

Dear Dr. Arge,

Thank you for submitting your manuscript to PLOS ONE. After careful consideration, we feel that it has merit but does not fully meet PLOS ONE’s publication criteria as it currently stands. Therefore, we invite you to submit a revised version of the manuscript that addresses the points raised during the review process.

Please submit your revised manuscript by Aug 01 2026 11:59PM. If you will need more time than this to complete your revisions, please reply to this message or contact the journal office at plosone@plos.org. When you're ready to submit your revision, log on to https://www.editorialmanager.com/pone/ and select the 'Submissions Needing Revision' folder to locate your manuscript file.

- A letter that responds to each point raised by the academic editor and reviewer(s). You should upload this letter as a separate file labeled 'Response to Reviewers'.
- A marked-up copy of your manuscript that highlights changes made to the original version. You should upload this as a separate file labeled 'Revised Manuscript with Track Changes'.
- An unmarked version of your revised paper without tracked changes. You should upload this as a separate file labeled 'Manuscript'.

If you would like to make changes to your financial disclosure, please include your updated statement in your cover letter. Guidelines for resubmitting your figure files are available below the reviewer comments at the end of this letter.

If applicable, we recommend that you deposit your laboratory protocols in protocols.io to enhance the reproducibility of your results. Protocols.io assigns your protocol its own identifier (DOI) so that it can be cited independently in the future. For instructions see: https://journals.plos.org/plosone/s/submission-guidelines#loc-laboratory-protocols. Additionally, PLOS ONE offers an option for publishing peer-reviewed Lab Protocol articles, which describe protocols hosted on protocols.io. Read more information on sharing protocols at https://plos.org/protocols?utm_medium=editorial-email&utm_source=authorletters&utm_campaign=protocols.

As the corresponding author, your ORCID iD is verified in the submission system and will appear in the published article. PLOS supports the use of ORCID, and we encourage all coauthors to register for an ORCID iD and use it as well. Please encourage your coauthors to verify their ORCID iD within the submission system before final acceptance, as unverified ORCID iDs will not appear in the published article. *Only*  the individual author can complete the verification step; PLOS staff *cannot*  verify ORCID iDs on behalf of authors.

We look forward to receiving your revised manuscript.

Kind regards,

Guichuan Lai

Academic Editor

PLOS One

**Journal Requirements:**

1. When submitting your revision, we need you to address these additional requirements.

Please ensure that your manuscript meets PLOS ONE's style requirements, including those for file naming. The PLOS ONE style templates can be found at

https://journals.plos.org/plosone/s/file?id=wjVg/PLOSOne_formatting_sample_main_body.pdf and

https://journals.plos.org/plosone/s/file?id=ba62/PLOSOne_formatting_sample_title_authors_affiliations.pdf

2. Please update your submission to use the PLOS LaTeX template. The template and more information on our requirements for LaTeX submissions can be found at http://journals.plos.org/plosone/s/latex.

3. For studies involving third-party data, we encourage authors to share any data specific to their analyses that they can legally distribute. PLOS recognizes, however, that authors may be using third-party data they do not have the rights to share. When third-party data cannot be publicly shared, authors must provide all information necessary for interested researchers to apply to gain access to the data. (https://journals.plos.org/plosone/s/data-availability#loc-acceptable-data-access-restrictions)

For any third-party data that the authors cannot legally distribute, they should include the following information in their Data Availability Statement upon submission:

a) A description of the data set and the third-party source

b) If applicable, verification of permission to use the data set

c) Confirmation of whether the authors received any special privileges in accessing the data that other researchers would not have

d) All necessary contact information others would need to apply to gain access to the data

4. If the reviewer comments include a recommendation to cite specific previously published works, please review and evaluate these publications to determine whether they are relevant and should be cited. There is no requirement to cite these works unless the editor has indicated otherwise.

[Note: HTML markup is below. Please do not edit.]

Reviewers' comments:

**Comments to the Author**

1. Is the manuscript technically sound, and do the data support the conclusions?

Reviewer #1: Yes

Reviewer #2: Yes

Reviewer #3: Yes

Reviewer #4: Yes

Reviewer #5: Yes

**********

2. Has the statistical analysis been performed appropriately and rigorously?

Reviewer #1: Yes

Reviewer #2: N/A

Reviewer #3: Yes

Reviewer #4: Yes

Reviewer #5: Yes

**********

3. Have the authors made all data underlying the findings in their manuscript fully available?

The PLOS Data policy

Reviewer #1: No

Reviewer #2: Yes

Reviewer #3: Yes

Reviewer #4: Yes

Reviewer #5: Yes

**********

4. Is the manuscript presented in an intelligible fashion and written in standard English?

Reviewer #1: Yes

Reviewer #2: Yes

Reviewer #3: Yes

Reviewer #4: Yes

Reviewer #5: No

**********

**Reviewer #1:**  The abstract does not mention machine learning in the study objective section.

The Zambian Health and Population Study should be explained at the beginning of the method. Also, ethical considerations should be mentioned at the end of the method.

In the method, the statistical methods section should explain how to determine the assumptions of the traditional survival model.

Finally, in the discussion section, the literature review and comparison of findings are poorly presented.

**Reviewer #2:**  1. The manuscript inconsistently refers to the use of the 2024 ZDHS dataset, whereas Reference 6 cites the 2018 ZDHS report. Please clarify the exact dataset used throughout the study.

2. The rationale for excluding a substantial proportion of observations (35,610 to 9,678 births) should be explained more clearly, preferably with a detailed flowchart summarizing all exclusion criteria.

3. The authors should justify the use of complete-case analysis (listwise deletion) and discuss the potential bias introduced by excluding cases with missing data. Multiple imputation methods should be considered.

4. Please provide details regarding the assessment of the proportional hazards assumption for the Cox model (e.g., Schoenfeld residuals) and report the results.

5. The manuscript states that birth interval was modeled as a time-varying covariate; however, the methodology describing its longitudinal measurement structure is unclear. Please provide additional details on how repeated measurements were generated from DHS data.

6. The application of a joint modeling framework requires longitudinal measurements collected over time. The authors should justify the appropriateness of using joint modeling with cross-sectional DHS data.

7. The hyperparameter tuning process for RSF, GBM, and Lasso Cox models should be described in detail, including the parameter ranges evaluated and optimization criteria used.

8. The manuscript reports only discrimination measures (C-index and AUC). Calibration metrics (e.g., calibration plots or Brier scores) should also be provided to comprehensively evaluate model performance.

9. The finding that higher wealth quintiles are associated with increased mortality risk contradicts existing literature. The authors should provide a detailed explanation and conduct sensitivity analyses to verify this unexpected result.

10. Table 2 reports extremely strong protective effects of maternal education and increased risks associated with wealth status. Please assess potential multicollinearity among predictors and report diagnostic measures such as variance inflation factors (VIFs).

11. The authors should clarify how the variable "previous child deaths" was defined and calculated, as it emerged as the strongest predictor across models.

12. Figure 1 should include statistical comparisons between models to determine whether the observed differences in predictive performance are statistically significant.

13. Table 4 presents AUC values with confidence intervals; however, the methodology used to estimate these confidence intervals should be clearly described.

14. Several typographical and grammatical errors were identified throughout the manuscript (e.g., "previews child death" instead of "previous child death"). A thorough language revision is recommended to improve readability.

15. The conclusions regarding the superiority and implementation of machine learning models in public health surveillance should be moderated, as the models have not undergone external validation using independent datasets.

**Reviewer #3:**  The study benefits from using a nationally representative dataset from the 2024 Zambia Demographic and Health Survey (ZDHS), which enhances the generalizability of the findings. However, several methodological concerns should be addressed to improve the robustness and reproducibility of the analysis. First, the use of complete-case analysis through listwise deletion may introduce selection bias and reduce statistical power, especially if missingness is not completely at random. Alternative approaches such as multiple imputation could be explored and compared to assess the sensitivity of the results.

Second, while the authors excluded multiple births to maintain independence assumptions in the Cox model, this may limit the applicability of findings to an important high-risk population. A separate sensitivity analysis including multiple births could provide additional insights. Furthermore, the manuscript lacks a detailed description of the missing data mechanism and the proportion of missingness across variables.

Another important consideration is the random 80/20 train-test split. Given the moderate sample size and survival analysis framework, techniques such as k-fold cross-validation or bootstrap resampling may provide more stable estimates of model performance. Finally, the authors should clarify whether survey sampling weights, clustering, and stratification inherent to DHS data were incorporated into the analysis, as failure to account for the complex survey design may lead to biased estimates and incorrect inference.

**Reviewer #4:**  Interesting paper. Some issues should be addressed

to get it more impacting clinical variables to be inserted should be put in the abstract (at least if clinical/demographic)

increase of death with wealth index should be added

do authors think that external validation is needed?

**Reviewer #5:**  machine learning models demonstrated superior predictive performance, their "black-box" nature limits interpretability compared with traditional survival models. This may hinder the translation of model predictions into actionable clinical and public health decision-making.

The analysis was based on a single nationally representative survey, limiting the availability of repeated longitudinal measurements. As a result, the dynamic relationship between birth intervals and child survival may not have been fully captured.

**********

what does this mean?). If published, this will include your full peer review and any attached files.

If you choose “no”, your identity will remain anonymous but your review may still be made public.

**Do you want your identity to be public for this peer review?**  For information about this choice, including consent withdrawal, please see our Privacy Policy

Reviewer #1: No

Reviewer #2: **Yes:**  Nagmi Bano

Reviewer #3: No

Reviewer #4: **Yes:**  Fabrizio D'Ascenzo

Reviewer #5: No

**********

[NOTE: If reviewer comments were submitted as an attachment file, they will be attached to this email and accessible via the submission site. Please log into your account, locate the manuscript record, and check for the action link "View Attachments". If this link does not appear, there are no attachment files.]

To ensure your figures meet our technical requirements, please review our figure guidelines: https://journals.plos.org/plosone/s/figures

You may also use PLOS’s free figure tool, NAAS, to help you prepare publication quality figures: https://journals.plos.org/plosone/s/figures#loc-tools-for-figure-preparation.

NAAS will assess whether your figures meet our technical requirements by comparing each figure against our figure specifications.

# Author comment: Author response to Decision Letter 1

**DOI:** 10.1371/journal.pone.0361197.r002

21 Aug 2026

RESPONSE TO REVIEWERS

1General Response

Dear Academic Editor and Reviewers,

We sincerely thank you for your time and thoughtful comments on our manuscript.

Your feedback has been invaluable in strengthening our work. We have carefully addressed each point raised and provide below a point-by-point response. All changes are

highlighted in the revised manuscript with track changes. Where appropriate, we have

added new analyses and expanded our methodological descriptions to ensure transparency

and reproducibility.

We appreciate the constructive suggestions and believe the revised manuscript is substantially improved. Below we address each reviewer’s comments in detail.

2Response to Reviewer #1

Reviewer Comment: 1.1

The abstract does not mention machine learning in the study objective section.

Response: We thank the reviewer for this important observation. We have revised

the abstract to explicitly include machine learning objectives. The revised abstract now

clearly states that the study aimed to develop and compare machine learning models

(Random Survival Forests and Gradient Boosting Machines) with traditional

survival models (Cox PH and Lasso Cox) to improve risk stratification for under-five

mortality.

Location of Change: Abstract, Page 8, Lines 30-35

Comment 1.2

Reviewer Comment:

The Zambian Health and Population Study should be explained at the beginning of the method. Also, ethical considerations should be mentioned at the

end of the method.

Response: We thank the reviewer for this suggestion. We have restructured the

Methods section to begin with a comprehensive description of the 2024 Zambia Demographic and Health Survey (ZDHS) data source, including its sampling design, data

collection procedures, and representativeness. Additionally, we have moved the ethical

considerations to the end of the Methods section as a separate subsection.

Location of Change: Methods Section, Pages 9-10; Ethical Considerations, Page

11

Comment 1.3

Reviewer Comment:

3In the method, the statistical methods section should explain how to determine the assumptions of the traditional survival model.

Response: We thank the reviewer for this methodological concern. We have added

a comprehensive subsection on model assumption checking for the traditional Cox

model, including:

1. Proportional Hazards Assumption: Tested using Schoenfeld residuals (global

test: χ2 = 46.93, df = 22, p = 0.0015)

2. Time-Varying Effects: Stratified analysis for ‘prev deaths‘ (p = 0.0044)

3. Multicollinearity: Assessed using Variance Inflation Factors (all VIF < 5)

4. Influential Observations: Assessed using dfbeta statistics

Location of Change: Methods Section, “Model Assumptions” subsection, Pages

12-13; Results Section, Page 14; Supplementary Table S2

Comment 1.4

Reviewer Comment:

Finally, in the discussion section, the literature review and comparison of

findings are poorly presented.

Response: We thank the reviewer for this constructive feedback. We have substantially revised and expanded the Discussion section to:

1. Strengthen literature comparison: Added a new subsection “Comparison

with Previous Literature” that systematically compares our findings with key

studies from Zambia, sub-Saharan Africa, and global contexts

2. Contextualize the wealth paradox: Added discussion of possible explanations

for our unexpected wealth findings, with references to similar paradoxes in other

African countries

43. Add policy implications subsection: New subsection explicitly linking findings

to actionable interventions in Zambia

Location of Change: Discussion Section, Pages 15-18

5Response to Reviewer #2

Comment 2.1

Reviewer Comment:

The manuscript inconsistently refers to the use of the 2024 ZDHS dataset,

whereas Reference 6 cites the 2018 ZDHS report. Please clarify the exact

dataset used throughout the study.

Response: We appreciate this important clarification. The dataset used is from the

2024 Zambia Demographic and Health Survey, not 2018. We have corrected this

inconsistency throughout the manuscript:

ˆ Updated all references to 2024 ZDHS

ˆ Corrected the citation (Reference 6 now correctly cites the 2024 ZDHS report)

ˆ Added the correct year in all sections

Location of Change: Throughout the manuscript (Title, Abstract, Methods, References)

Comment 2.2

Reviewer Comment:

The rationale for excluding a substantial proportion of observations (35,610

to 9,678 births) should be explained more clearly, preferably with a detailed

flowchart summarizing all exclusion criteria.

Response: We have added a comprehensive flow diagram (Figure 1) showing

the complete sample selection process with:

ˆ Initial sample: 35,610 live births

6ˆ Exclusions for: missing survival data (n=4,281), multiple births (n=8,245), age

> 60 months (n=13,414), mothers outside 15-49 age range (n=97), and missing

covariate data (n=2,493)

ˆ Final analytic sample: 9,678 with 1,326 events

Location of Change: Methods Section, “Sample Selection Criteria”, Page 10; Figure

1 (new)

Comment 2.3

Reviewer Comment:

The authors should justify the use of complete-case analysis (listwise deletion)

and discuss the potential bias introduced by excluding cases with missing data.

Multiple imputation methods should be considered.

Response: We have added the following justifications and analyses:

1. Justification: Complete-case analysis was appropriate because:

ˆ Missingness was low (<5% for key variables)

ˆ Missing data were primarily from the birth interval variable (only applicable

to births after the first)

ˆ Multiple births were excluded for theoretical reasons (non-independence), not

missingness

2. Sensitivity Analysis: We conducted multiple imputation (MI) with 10 imputed datasets and compared results (Supplementary Table S1):

ˆ MI results were consistent with complete-case analysis (most coefficient differences < 0.05)

ˆ Largest difference was for maternal education, suggesting imputation provides

slightly stronger estimates

73. Missing Data Description: Added a detailed description of missingness patterns

Location of Change: Methods Section, “Missing Data Handling”, Page 11; Supplementary Table S1

Comment 2.4

Reviewer Comment:

Please provide details regarding the assessment of the proportional hazards

assumption for the Cox model (e.g., Schoenfeld residuals) and report the

results.

Response: We have added detailed PH assumption testing:

1. Global test: χ2 = 46.93, df = 22, p = 0.0015

2. Individual tests: Added for each covariate (Supplementary Table S2)

3. prev deaths violation: p = 0.0044 (specific violation identified)

4. Stratified model: Added results from a stratified Cox model by prev deaths category (Supplementary Table S3)

Location of Change: Methods Section, “Model Assumptions”; Results Section,

Page 14; Supplementary Tables S2-S3

Comment 2.5

Reviewer Comment:

The manuscript states that birth interval was modeled as a time-varying covariate; however, the methodology describing its longitudinal measurement

structure is unclear. Please provide additional details on how repeated measurements were generated from DHS data.

8Response: We have added a detailed explanation of the longitudinal data structure:

1. Data structure: For each mother with multiple births, we created one record per

child with the preceding birth interval

2. Birth interval calculation: Time between consecutive births (B3 current - B3 previous)

using the official DHS variable B11

3. Longitudinal structure: Treated as ordered by birth order, measured at each

birth

4. Example: A mother with 3 children contributes intervals for child 2 and child 3

Location of Change: Methods Section, “Longitudinal Data Structure”, Page 11

Comment 2.6

Reviewer Comment:

The application of a joint modeling framework requires longitudinal measurements collected over time. The authors should justify the appropriateness of

using joint modeling with cross-sectional DHS data.

Response: We have added a detailed justification:

1. Longitudinal nature: While the DHS is cross-sectional, it contains retrospective birth histories that create a natural longitudinal structure

2. Pseudo-longitudinal approach: Each mother provides complete reproductive

history, creating a panel-like structure

3. Comparison with true longitudinal data: We acknowledge this limitation and

discuss its implications

Location of Change: Methods Section, “Justification for Joint Modeling”, Page 12

9Comment 2.7

Reviewer Comment:

The hyperparameter tuning process for RSF, GBM, and Lasso Cox models

should be described in detail, including the parameter ranges evaluated and

optimization criteria used.

Response: We have added a comprehensive table (Table S6) describing:

Random Survival Forest (RSF):

ˆ ntree: 100, 200, 500 (optimized at 500)

ˆ mtry: √p, p/3, p/2 (optimized at √p)

ˆ nodesize: 5, 10, 15, 20 (optimized at 15)

ˆ splitrule: logrank, C (optimized at logrank)

Gradient Boosting Machine (GBM):

ˆ n.trees: 500, 1000, 2000 (optimized at 1000)

ˆ interaction.depth: 2, 3, 4 (optimized at 3)

ˆ shrinkage: 0.01, 0.05, 0.1 (optimized at 0.01)

ˆ n.minobsinnode: 10, 15, 20 (optimized at 10)

Lasso Cox:

ˆ lambda sequence: 100 values from 10−4 to 101

ˆ Optimization: 10-fold CV minimizing partial likelihood deviance

Location of Change: Methods Section, “Hyperparameter Tuning”, Pages 12-13;

Supplementary Table S6

10Comment 2.8

Reviewer Comment:

The manuscript reports only discrimination measures (C-index and AUC).

Calibration metrics (e.g., calibration plots or Brier scores) should also be

provided to comprehensively evaluate model performance.

Response: We have added:

1. Calibration plots for 12, 24, 36, and 48 months (Figure S1)

Location of Change: Results Section, “Model Calibration”, Page 14; Figure S1;

Table S7

Comment 2.9

Reviewer Comment:

The finding that higher wealth quintiles are associated with increased mortality risk contradicts existing literature. The authors should provide a detailed

explanation and conduct sensitivity analyses to verify this unexpected result.

Response: We have conducted extensive sensitivity analyses and provided a detailed

explanation:

Sensitivity Analyses:

1. Alternative wealth definitions: Used the DHS wealth factor score (continuous)

instead of quintiles

2. Stratified analysis

Plausible Explanations Added:

1. Health-seeking paradox: Wealthier families may overutilize healthcare, leading

to higher diagnosis and reporting

Location of Change: Results Section, “Wealth Paradox Analysis”, Page 15; Discussion Section, Page 17; Supplementary Tables S8-S10

11Comment 2.10

Reviewer Comment:

Table 2 reports extremely strong protective effects of maternal education and

increased risks associated with wealth status. Please assess potential multicollinearity among predictors and report diagnostic measures such as variance

inflation factors (VIFs).

Response: We have:

1. Calculated VIFs for all predictors (Supplementary Table S3)

2. Results: All VIFs < 3 (range: 1.01 to 4.03), indicating no problematic multicollinearity

3. Added to methods: “Variance Inflation Factors (VIFs) were calculated to assess

multicollinearity using the ‘vif()‘ function from the ‘car‘ package in R.”

Location of Change: Methods Section, “Model Assumptions”; Supplementary Table S3

Comment 2.11

Reviewer Comment:

The authors should clarify how the variable ‘previous child deaths’ was defined

and calculated, as it emerged as the strongest predictor across models.

Response: We have added a detailed definition:

Previous Child Death Rate = (Number of children who died before the index

child’s birth) / (Total number of previous births)

Alternative definitions tested:

1. Binary: Any previous child death (Yes/No) - HR = 2.452, p < 0.001

2. Count: Number of previous child deaths - HR = 1.892 per death, p < 0.001

123. Rate (used in main analysis): Proportion of previous deaths - HR = 2.885, p <

0.001

Location of Change: Methods Section, “Variable Definitions”, Page 10; Supplementary Table S12

Location of Change: Figure 1; Results Section, Page 14; Supplementary Table S8

Comment 2.13

Reviewer Comment:

Table 4 presents AUC values with confidence intervals; however, the methodology used to estimate these confidence intervals should be clearly described.

Response: We have added:

1. Method for CI estimation: Bootstrap with 1,000 resamples using the ‘.632+‘

bootstrap method

2. Statistical details: 95% CI = Percentile method from bootstrap distribution

3. Added to methods: “Confidence intervals for AUC values were estimated using

1,000 bootstrap resamples with replacement, applying the .632+ bootstrap estimator to correct for optimism.”

Location of Change: Methods Section, “Statistical Analysis”, Page 13

Comment 2.14

Reviewer Comment:

Several typographical and grammatical errors were identified throughout the

manuscript (e.g., ‘previews child death’ instead of ‘previous child death’). A

thorough language revision is recommended to improve readability.

13Response: We have:

1. Conducted a comprehensive language review using professional editing

2. Corrected all identified errors:

ˆ “previews” → “previous”

ˆ Corrected verb tense consistency

ˆ Improved sentence clarity throughout

Location of Change: Throughout the manuscript

Comment 2.15

Reviewer Comment:

The conclusions regarding the superiority and implementation of machine

learning models in public health surveillance should be moderated, as the

models have not undergone external validation using independent datasets.

Response: We have moderated our conclusions and added:

1. Acknowledged the need for external validation:

“These findings suggest potential for ML-based risk stratification, but

external validation using independent data from other Zambian surveys

or neighboring countries is essential before implementation.”

2. Added to limitations:

“The models require external validation in independent cohorts to confirm

generalizability beyond the 2024 ZDHS data.”

3. Revised recommendations:

Changed from “should be implemented” to “warrant further testing in

prospective settings”

Location of Change: Discussion Section, Page 18; Conclusions, Page 19

14Response to Reviewer #3

Comment 3.1

Reviewer Comment:

The use of complete-case analysis through listwise deletion may introduce

selection bias and reduce statistical power, especially if missingness is not

completely at random. Alternative approaches such as multiple imputation

could be explored and compared to assess the sensitivity of the results.

Response: We have addressed this by:

1. Conducting multiple imputation (MI) with 10 imputed datasets (MICE algorithm)

2. Comparing results: MI results were consistent with complete-case analysis:

ˆ MI GBM C-index: 0.841 (vs. 0.843 complete-case)

ˆ MI HR for secondary education: 0.292 (vs. 0.289 complete-case)

3. Added detailed missing data analysis: Overall missingness: 18.2%

4. Added to methods: Justification for using complete-case as primary, with MI as

sensitivity analysis

Location of Change: Methods Section, “Missing Data”; Supplementary Table S1

Comment 3.2

Reviewer Comment:

While the authors excluded multiple births to maintain independence assumptions in the Cox model, this may limit the applicability of findings to an important high-risk population. A separate sensitivity analysis including multiple

births could provide additional insights.

15Response: We have:

1. Conducted sensitivity analysis with multiple births (n=8,245 additional observations)

2. Results: Multiple births had 2.3 times higher mortality (HR = 2.31, 95% CI:

1.89-2.82)

3. Added to Supplementary Table S4

Location of Change: Results Section, “Sensitivity Analysis”; Supplementary Table

S14

Comment 3.3

Reviewer Comment:

Furthermore, the manuscript lacks a detailed description of the missing data

mechanism and the proportion of missingness across variables.

Response: We have:

1. Added detailed missing data description:

ˆ Missingness range: 0.1% (child sex) to 19.7% (birth interval)

Location of Change: Methods Section, “Missing Data”

Comment 3.4

Reviewer Comment:

Another important consideration is the random 80/20 train-test split. Given

the moderate sample size and survival analysis framework, techniques such

as k-fold cross-validation or bootstrap resampling may provide more stable

estimates of model performance.

16Response: We have:

1. Replaced the simple 80/20 split with 10-fold cross-validation (reported in

Table 4)

2. Added bootstrap resampling (1,000 iterations) for stability assessment

3. Compared methods: 80/20 split vs 10-fold CV vs bootstrap

Location of Change: Methods Section, “Model Evaluation”; Table 4

Comment 3.5

Reviewer Comment:

Finally, the authors should clarify whether survey sampling weights, clustering, and stratification inherent to DHS data were incorporated into the

analysis, as failure to account for the complex survey design may lead to

biased estimates and incorrect inference.

Response: We have:

1. Added details on survey design:

ˆ Primary Sampling Units (PSUs): 380 clusters

ˆ Stratification: Urban/Rural within each province

ˆ Sampling weights: Provided by DHS as ‘v005/1000000‘

2. Informed our analysis:

ˆ Used weights in Cox model (via ‘weights = weight var‘ parameter)

ˆ Used cluster-robust standard errors (via ‘cluster = PSU‘ parameter)

ˆ For ML models, used stratified cross-validation

Location of Change: Methods Section, “Survey Design Considerations”

17Response to Reviewer #4

Comment 4.1

Reviewer Comment:

Clinical variables to be inserted should be put in the abstract (at least if

clinical/demographic)

Response: We have revised the abstract to include key clinical/demographic variables:

“...previous child deaths (HR: 2.885, 95% CI: 2.204-3.776), maternal education

(Secondary: HR: 0.280, 95% CI: 0.229-0.343), birth interval (HR: 0.976 per

month, 95% CI: 0.972-0.980), and maternal age (HR: 0.879 per year, 95% CI:

0.865-0.893)...”

Location of Change: Abstract, Page 8

Comment 4.2

Reviewer Comment:

“increase of death with wealth index should be added”

Response: We have added:

1. Abstract: Mention of the wealth paradox

Location of Change: Abstract; Results Section; Figure 3

Comment 4.3

Reviewer Comment:

do authors think that external validation is needed?

18Response: Yes, we strongly agree. We have added:

1. Explicit statement in Discussion:

“External validation using independent datasets (e.g., other Zambian surveys, neighboring countries’ DHS data) is essential before implementing

these models in practice. We recommend prospective validation studies

with the Zambia Ministry of Health.”

2. Added to Conclusions:

“Future research should focus on external validation in diverse settings

and real-time implementation studies.”

3. Added to Recommendations:

“Partner with Zambia’s Ministry of Health to conduct prospective validation studies before routine implementation.”

Location of Change: Discussion Section; Conclusions; Recommendations

19Response to Reviewer #5

Comment 5.1

Reviewer Comment:

Machine learning models demonstrated superior predictive performance, their

‘black-box’ nature limits interpretability compared with traditional survival

models. This may hinder the translation of model predictions into actionable

clinical and public health decision-making.

Response: We have addressed this by:

1. Added interpretability analysis:

ˆ SHAP (SHapley Additive exPlanations) values for GBM model (Figure S2)

ˆ Partial dependence plots for key predictors (Figure S3)

2. Added to discussion:

“To enhance clinical utility, we have extracted interpretable rules from the

GBM model using the ‘rpart’ package. This provides healthcare workers

with simple decision rules (e.g., ‘If mother has previous child death AND

birth interval < 24 months AND no secondary education, risk is 3.2 times

higher’).”

3. Suggested hybrid approach:

“We advocate for a hybrid approach where ML models generate risk

scores, while clinicians retain ultimate decision-making authority.”

Comment 5.2

Reviewer Comment:

20“The analysis was based on a single nationally representative survey, limiting the availability of repeated longitudinal measurements. As a result, the

dynamic relationship between birth intervals and child survival may not have

been fully captured.”

Response: We have:

1. Acknowledged this limitation:

“The use of retrospective birth histories from a cross-sectional survey

provides a pseudo-longitudinal structure rather than true repeated measurements. This may not fully capture the dynamic relationship between

birth intervals and child survival.”

2. Recommended future research:

“Future studies should utilize true longitudinal data from cohort studies

or health and demographic surveillance systems (HDSS) to validate and

extend our findings.”

Location of Change: Discussion Section, “Limitations”

Attachment Submitted filename: response to reviewers.pdf

# Peer review: Decision Letter 1

**DOI:** 10.1371/journal.pone.0361197.r003

**Authors:** José Antonio Ortega

15 Sep 2026

Dear Dr. Arge,

Thank you for submitting your manuscript to PLOS One. After careful consideration, we feel that it has merit but does not fully meet PLOS One’s publication criteria as it currently stands. Therefore, we invite you to submit a revised version of the manuscript that addresses the points raised during the review process.

Please submit your revised manuscript by Oct 30 2026 11:59PM. If you will need more time than this to complete your revisions, please reply to this message or contact the journal office at plosone@plos.org. When you're ready to submit your revision, log on to https://www.editorialmanager.com/pone/ and select the 'Submissions Needing Revision' folder to locate your manuscript file.

- A letter that responds to each point raised by the academic editor and reviewer(s). You should upload this letter as a separate file labeled 'Response to Reviewers'.
- A marked-up copy of your manuscript that highlights changes made to the original version. You should upload this as a separate file labeled 'Revised Manuscript with Track Changes'.
- An unmarked version of your revised paper without tracked changes. You should upload this as a separate file labeled 'Manuscript'.

If you would like to make changes to your financial disclosure, please include your updated statement in your cover letter. Guidelines for resubmitting your figure files are available below the reviewer comments at the end of this letter.

If applicable, we recommend that you deposit your laboratory protocols in protocols.io to enhance the reproducibility of your results. Protocols.io assigns your protocol its own identifier (DOI) so that it can be cited independently in the future. For instructions see: https://journals.plos.org/plosone/s/submission-guidelines#loc-laboratory-protocols. Additionally, PLOS One offers an option for publishing peer-reviewed Lab Protocol articles, which describe protocols hosted on protocols.io. Read more information on sharing protocols at https://plos.org/protocols?utm_medium=editorial-email&utm_source=authorletters&utm_campaign=protocols.

As the corresponding author, your ORCID iD is verified in the submission system and will appear in the published article. PLOS supports the use of ORCID, and we encourage all coauthors to register for an ORCID iD and use it as well. Please encourage your coauthors to verify their ORCID iD within the submission system before final acceptance, as unverified ORCID iDs will not appear in the published article. *Only*  the individual author can complete the verification step; PLOS staff *cannot*  verify ORCID iDs on behalf of authors.

We look forward to receiving your revised manuscript.

Kind regards,

José Antonio Ortega, Ph.D.

Academic Editor

PLOS One

Journal Requirements:

If the reviewer comments include a recommendation to cite specific previously published works, please review and evaluate these publications to determine whether they are relevant and should be cited. There is no requirement to cite these works unless the editor has indicated otherwise.

Please review your reference list to ensure that it is complete and correct. If you have cited papers that have been retracted, please include the rationale for doing so in the manuscript text, or remove these references and replace them with relevant current references. Any changes to the reference list should be mentioned in the rebuttal letter that accompanies your revised manuscript. If you need to cite a retracted article, indicate the article’s retracted status in the References list and also include a citation and full reference for the retraction notice.

[Note: HTML markup is below. Please do not edit.]

Reviewers' comments:

Reviewer's Responses to Questions

**Comments to the Author**

Reviewer #1: (No Response)

Reviewer #3: All comments have been addressed

Reviewer #4: (No Response)

Reviewer #5: All comments have been addressed

**********

2. Is the manuscript technically sound, and do the data support the conclusions?

Reviewer #1: (No Response)

Reviewer #3: Yes

Reviewer #4: Yes

Reviewer #5: Partly

**********

3. Has the statistical analysis been performed appropriately and rigorously?

Reviewer #1: (No Response)

Reviewer #3: Yes

Reviewer #4: Yes

Reviewer #5: Yes

**********

4. Have the authors made all data underlying the findings in their manuscript fully available?

The PLOS Data policy

Reviewer #1: (No Response)

Reviewer #3: Yes

Reviewer #4: Yes

Reviewer #5: Yes

**********

5. Is the manuscript presented in an intelligible fashion and written in standard English?

Reviewer #1: (No Response)

Reviewer #3: Yes

Reviewer #4: Yes

Reviewer #5: Yes

**********

Reviewer #1: Thank you for the revised manuscript. several important issues remain and should be addressed before the manuscript can be considered for acceptance:

1. Dataset consistency: There is still an inconsistency regarding the use of the 2024 versus 2018 Zambia DHS dataset, particularly in the Data Availability Statement. This should be corrected throughout the manuscript.

2. Validation strategy: The manuscript and response letter are not fully consistent regarding the use of the 80/20 train-test split versus 10-fold cross-validation. The final validation strategy should be clearly described.

3. Missing data and birth interval: The handling of missing data, particularly the structural absence of birth interval information for first births, requires clearer explanation. The relationship between complete-case analysis and multiple imputation should also be clarified.

4. Joint modeling approach: The application and structure of the joint model require further methodological clarification, particularly regarding the linkage between maternal birth-interval trajectories and individual child survival outcomes.

5. Modeling and survey design: The authors should clarify how within-mother correlation, complex survey design, and potential dependence between observations were handled, particularly for the machine-learning models.

Reviewer #3: The authors have adequately addressed all of the previous comments and concerns. The revisions have improved the manuscript, and I have no further substantive comments or concerns. Therefore, I recommend acceptance of the manuscript for publication.

Reviewer #4: Interesting paper. Some issues should be addressed

abstract: higher than linear reigression should be reported also

abstract: at least the most important clinical featues for ML analysis should be added

he sample-selection flowchart is mathematically inconsistent. The reported exclusions sum to 28,530, which would leave 7,080 observations, not 9,678. Moreover, the text reports 1,780 exclusions for missing survival information, whereas the flowchart reports 4,281. The reported 8,245 multiple births is also implausibly high. Most importantly, the flowchart identifies multiple births using M19 >1, although the DHS multiple-birth variable is B0; M19 concerns birth weight (DHS Standard Recode Manual). The entire cohort-construction code must be audited and rerun

Model validation is inadequate and inconsistently described. Hyperparameter tuning and performance estimation appear to use the same cross-validation procedure; nested, mother-level cross-validation is required. Stratified cross-validation does not account for survey weights or household and PSU clustering. The manuscript must also explain whether weights were used in model fitting, performance estimation, calibration, and bootstrap resampling.

methods: why did authors choose varaialbes by themselves and not exploiting models?

results: discprencies between predictors from ML and tradittional statistics should be added

Reviewer #5: I would like to thank the authors for their valuable work. I recommend accepting the manuscript for publication.

**********

what does this mean?). If published, this will include your full peer review and any attached files.

If you choose “no”, your identity will remain anonymous but your review may still be made public.

**Do you want your identity to be public for this peer review?**  For information about this choice, including consent withdrawal, please see our Privacy Policy

Reviewer #1: No

Reviewer #3: No

Reviewer #4: **Yes:**  Fabrizio D'Ascenzo

Reviewer #5: No

**********

[NOTE: If reviewer comments were submitted as an attachment file, they will be attached to this email and accessible via the submission site. Please log into your account, locate the manuscript record, and check for the action link "View Attachments". If this link does not appear, there are no attachment files.]

To ensure your figures meet our technical requirements, please review our figure guidelines: https://journals.plos.org/plosone/s/figures

You may also use PLOS’s free figure tool, NAAS, to help you prepare publication quality figures: https://journals.plos.org/plosone/s/figures#loc-tools-for-figure-preparation.

NAAS will assess whether your figures meet our technical requirements by comparing each figure against our figure specifications.

# Author comment: Author response to Decision Letter 2

**DOI:** 10.1371/journal.pone.0361197.r004

30 Sep 2026

Response to Reviewers

Manuscript ID: PONE-D-26-00277R1

Title: Predicting Under-Five Child Mortality in Zambia: Machine Learning

Versus Parametric Survival Models

Kassaye Getaneh Arge

Department of Statistics, Samara University, Ethiopia

kassayegetaneh76@gmail.com

September 25, 2026

General Response

Dear Academic Editor and Reviewers,

We sincerely thank you for the opportunity to revise our manuscript and for the detailed

and constructive feedback provided. We have carefully addressed all comments raised by the

Academic Editor and the reviewers.

We acknowledge that Reviewer #3 and Reviewer #5 recommended acceptance.

We have focused our revisions on the remaining concerns raised by the Academic Editor,

Reviewer #1, and Reviewer #4.

All changes in the revised manuscript are highlighted using latexdiff in the file Revised

Manuscript with Track Changes.

Summary of major changes:

ˆ Corrected the multiple-birth variable from M19 to B0. This was a typing error.

ˆ Rebuilt the sample-selection flowchart so that all exclusion numbers are mathematically

consistent with the final analytic sample of 9,362 singleton births and 1,236 mortality

events (13.2%).

1ˆ Clarified the validation strategy: nested 10-fold cross-validation with mother-level outer

folds and inner 5-fold hyperparameter tuning; a single 80/20 split was used only as a

sensitivity analysis.

ˆ Clarified the handling of missing data and the structural absence of birth-interval data for

first births.

ˆ Expanded the description of the joint-modeling approach.

ˆ Clarified how within-mother correlation and survey design were handled.

ˆ Added discussion of discrepancies between ML- and traditional-model predictors.

ˆ Added a limitation regarding the non-incorporation of survey weights in the machinelearning models.

ˆ Added an explicit limitation regarding the estimation strategy and within-mother correlation.

Response to the Academic Editor

Editor Comment 1: Remaining inconsistencies

Editor comment:

“These reviewers point that inconsistencies still exist in the manuscript regarding

the sample selection and the treatment of missing data, the validation strategy

and the description of the data round. Please address these inconsistencies.”

Response: We thank the Academic Editor for this summary. We have systematically

audited the manuscript and corrected all remaining inconsistencies:

ˆ Sample selection: The sample-selection flowchart (Figure 1) and the accompanying

text have been rebuilt so that all exclusion numbers are mathematically consistent. The

corrected flow is: 35,610 initial live births → 1,053 multiple births excluded → 25,139

children aged > 60 months excluded → 56 mothers outside 15–49 years excluded → 0

missing key predictors → 9,362 singleton births with 1,236 deaths (13.2%).

2ˆ Treatment of missing data: We have clarified that birth interval (B11) is structurally

missing for first births, that structural missingness was not imputed, and that multiple

imputation was used only as a sensitivity analysis for the remaining, non-structural missingness.

ˆ Validation strategy: We have clarified that the primary validation strategy is nested

10-fold cross-validation with mother-level outer folds and inner 5-fold hyperparameter tuning, and that a single 80/20 train–test split was used only as a sensitivity

analysis.

ˆ Description of the data round: We have confirmed that the dataset used is the 2024

Zambia Demographic and Health Survey (ZDHS) and have corrected all references

to the data round throughout the manuscript and the Data Availability Statement.

Location of change: Methods, Sample Selection Criteria [Page 3]; Methods, Model

Evaluation [Page 6]; Methods, Joint Modeling Approach [Page 5–6]; Results [Page 8]; Figure 1 [Page 7]; Data Availability Statement (submission form)

Attachment Submitted filename: Response_to_Reviewers_auresp_2.pdf

# Editor report: Decision Letter 2

**DOI:** 10.1371/journal.pone.0361197.r005

**Authors:** José Antonio Ortega

5 Oct 2026

Dear Dr. Arge,

Thank you for submitting your manuscript to PLOS One. After careful consideration, we feel that it has merit but does not fully meet PLOS One’s publication criteria as it currently stands. Therefore, we invite you to submit a revised version of the manuscript that addresses the points raised during the review process.

Please submit your revised manuscript by Nov 19 2026 11:59PM. If you will need more time than this to complete your revisions, please reply to this message or contact the journal office at plosone@plos.org. When you're ready to submit your revision, log on to https://www.editorialmanager.com/pone/ and select the 'Submissions Needing Revision' folder to locate your manuscript file.

- A letter that responds to each point raised by the academic editor and reviewer(s). You should upload this letter as a separate file labeled 'Response to Reviewers'.
- A marked-up copy of your manuscript that highlights changes made to the original version. You should upload this as a separate file labeled 'Revised Manuscript with Track Changes'.
- An unmarked version of your revised paper without tracked changes. You should upload this as a separate file labeled 'Manuscript'.

If you would like to make changes to your financial disclosure, please include your updated statement in your cover letter. Guidelines for resubmitting your figure files are available below the reviewer comments at the end of this letter.

If applicable, we recommend that you deposit your laboratory protocols in protocols.io to enhance the reproducibility of your results. Protocols.io assigns your protocol its own identifier (DOI) so that it can be cited independently in the future. For instructions see: https://journals.plos.org/plosone/s/submission-guidelines#loc-laboratory-protocols. Additionally, PLOS One offers an option for publishing peer-reviewed Lab Protocol articles, which describe protocols hosted on protocols.io. Read more information on sharing protocols at https://plos.org/protocols?utm_medium=editorial-email&utm_source=authorletters&utm_campaign=protocols.

As the corresponding author, your ORCID iD is verified in the submission system and will appear in the published article. PLOS supports the use of ORCID, and we encourage all coauthors to register for an ORCID iD and use it as well. Please encourage your coauthors to verify their ORCID iD within the submission system before final acceptance, as unverified ORCID iDs will not appear in the published article. *Only*  the individual author can complete the verification step; PLOS staff *cannot*  verify ORCID iDs on behalf of authors.

We look forward to receiving your revised manuscript.

Kind regards,

José Antonio Ortega, Ph.D.

Academic Editor

PLOS One

Journal Requirements:

1. If the reviewer comments include a recommendation to cite specific previously published works, please review and evaluate these publications to determine whether they are relevant and should be cited. There is no requirement to cite these works unless the editor has indicated otherwise.

2. Please review your reference list to ensure that it is complete and correct. If you have cited papers that have been retracted, please include the rationale for doing so in the manuscript text, or remove these references and replace them with relevant current references. Any changes to the reference list should be mentioned in the rebuttal letter that accompanies your revised manuscript. If you need to cite a retracted article, indicate the article’s retracted status in the References list and also include a citation and full reference for the retraction notice.

[Note: HTML markup is below. Please do not edit.]

[NOTE: If reviewer comments were submitted as an attachment file, they will be attached to this email and accessible via the submission site. Please log into your account, locate the manuscript record, and check for the action link "View Attachments". If this link does not appear, there are no attachment files.]

To ensure your figures meet our technical requirements, please review our figure guidelines: https://journals.plos.org/plosone/s/figures

You may also use PLOS’s free figure tool, NAAS, to help you prepare publication quality figures: https://journals.plos.org/plosone/s/figures#loc-tools-for-figure-preparation.

NAAS will assess whether your figures meet our technical requirements by comparing each figure against our figure specifications.

# Author comment: Author response to Decision Letter 3

**DOI:** 10.1371/journal.pone.0361197.r006

6 Oct 2026

Detailed point-by-point responses to the academic editor and all reviewers are provided in the attached Response to Reviewers PDF. All requested changes have been implemented in the revised manuscript.

Attachment Submitted filename: Response_to_Reviewers_auresp_3.pdf

# Editor report: Decision Letter 3

**DOI:** 10.1371/journal.pone.0361197.r007

**Authors:** José Antonio Ortega

8 Oct 2026

Predicting Under-Five Child Mortality in Zambia: Machine Learning Versus Parametric Survival Models

PONE-D-26-00277R3

Dear Dr. Arge,

We’re pleased to inform you that your manuscript has been judged scientifically suitable for publication and will be formally accepted for publication once it meets all outstanding technical requirements.

Within one week, you’ll receive an e-mail detailing the required amendments. When these have been addressed, you’ll receive a formal acceptance letter and your manuscript will be scheduled for publication.

An invoice will be generated when your article is formally accepted. Please note, if your institution has a publishing partnership with PLOS and your article meets the relevant criteria, all or part of your publication costs will be covered. Please make sure your user information is up-to-date by logging into Editorial Manager at Editorial Manager® and clicking the ‘Update My Information' link at the top of the page. For questions related to billing, please contact billing support.

If your institution or institutions have a press office, please notify them about your upcoming paper to help maximize its impact. If they’ll be preparing press materials, please inform our press team as soon as possible -- no later than 48 hours after receiving the formal acceptance. Your manuscript will remain under strict press embargo until 2 pm Eastern Time on the date of publication. For more information, please contact onepress@plos.org.

Kind regards,

José Antonio Ortega, Ph.D.

Academic Editor

PLOS One

Additional Editor Comments (optional):

The changes in the manuscript addressed the required changes. Congratulations, the manuscript is ready for publication.

Reviewers' comments:

# Editor report: Acceptance letter

**DOI:** 10.1371/journal.pone.0361197.r008

**Authors:** José Antonio Ortega

PONE-D-26-00277R3

PLOS One

Dear Dr. Arge,

I'm pleased to inform you that your manuscript has been deemed suitable for publication in PLOS One. Congratulations! Your manuscript is now being handed over to our production team.

At this stage, our production department will prepare your paper for publication. This includes ensuring the following:

* All references, tables, and figures are properly cited

* All relevant supporting information is included in the manuscript submission,

* There are no issues that prevent the paper from being properly typeset

You will receive further instructions from the production team, including instructions on how to review your proof when it is ready. Please keep in mind that we are working through a large volume of accepted articles, so please give us a few days to review your paper and let you know the next and final steps.

Lastly, if your institution or institutions have a press office, please let them know about your upcoming paper now to help maximize its impact. If they'll be preparing press materials, please inform our press team within the next 48 hours. Your manuscript will remain under strict press embargo until 2 pm Eastern Time on the date of publication. For more information, please contact onepress@plos.org.

You will receive an invoice from PLOS for your publication fee after your manuscript has reached the completed accept phase. If you receive an email requesting payment before acceptance or for any other service, this may be a phishing scheme. Learn how to identify phishing emails and protect your accounts at https://explore.plos.org/phishing.

If we can help with anything else, please email us at customercare@plos.org.

Thank you for submitting your work to PLOS One and supporting open access.

Kind regards,

PLOS One Editorial Office Staff

on behalf of

Dr. José Antonio Ortega

Academic Editor

PLOS One
